# Progesterone-induced progesterone receptor membrane component 1 rise-to-decline changes are essential for decidualization

**DOI:** 10.1186/s12958-024-01188-9

**Published:** 2024-02-03

**Authors:** Hailun Liu, André Franken, Alexandra P. Bielfeld, Tanja Fehm, Dieter Niederacher, Zhongping Cheng, Hans Neubauer, Nadia Stamm

**Affiliations:** 1 Department of Obstetrics and Gynecology, Life Science Center, University Hospital and Medical Faculty of the Heinrich-Heine University Duesseldorf, Duesseldorf, Germany; 2 Department of OB/GYN & REI, UniKiD, University Hospital and Faculty of Medicine, Heinrich Heine University, Duesseldorf, Germany; 3 Department of Obstetrics and Gynecology, Shanghai Tenth People’s Hospital, Tongji University School of Medicine, Shanghai, 200072 China; 4 Institute of Gynecological Minimally Invasive Medicine, Tongji University School of Medicine, Shanghai, 200072 China

**Keywords:** Decidualization, Progesterone receptor membrane component 1 (PGRMC1), Endometrium, Telomerase-immortalized human endometrial stromal cells (T-HESCs), Infertility, Prohibitin-1 (PHB1), Prohibitin-2 (PHB2), AG205, Rise-to-decline pattern

## Abstract

**Background:**

Decidualization of endometrial cells is the prerequisite for embryo implantation and subsequent placenta formation and is induced by rising progesterone levels following ovulation. One of the hormone receptors contributing to endometrial homeostasis is Progesterone Receptor Membrane Component 1 (PGRMC1), a non-classical membrane-bound progesterone receptor with yet unclear function. In this study, we aimed to investigate how PGRMC1 contributes to human decidualization.

**Methods:**

We first analyzed PGRMC1 expression profile during a regular menstrual cycle in RNA-sequencing datasets. To further explore the function of PGRMC1 in human decidualization, we implemented an inducible decidualization system, which is achieved by culturing two human endometrial stromal cell lines in decidualization-inducing medium containing medroxyprogesterone acetate and 8-Br-cAMP. In our system, we measured PGRMC1 expression during hormone induction as well as decidualization status upon PGRMC1 knockdown at different time points. We further conferred proximity ligation assay to identify PGRMC1 interaction partners.

**Results:**

In a regular menstrual cycle, PGRMC1 mRNA expression is gradually decreased from the proliferative phase to the secretory phase. In in vitro experiments, we observed that PGRMC1 expression follows a rise-to-decline pattern, in which its expression level initially increased during the first 6 days after induction (PGRMC1 increasing phase) and decreased in the following days (PGRMC1 decreasing phase). Knockdown of PGRMC1 expression before the induction led to a failed decidualization, while its knockdown after induction did not inhibit decidualization, suggesting that the progestin-induced ‘PGRMC1 increasing phase’ is essential for normal decidualization. Furthermore, we found that the interactions of prohibitin 1 and prohibitin 2 with PGRMC1 were induced upon progestin treatment. Knocking down each of the prohibitins slowed down the decidualization process compared to the control, suggesting that PGRMC1 cooperates with prohibitins to regulate decidualization.

**Conclusions:**

According to our findings, PGRMC1 expression followed a progestin-induced rise-to-decline expression pattern during human endometrial decidualization process; and the correct execution of this expression program was crucial for successful decidualization. Thereby, the results of our in vitro model explained how PGRMC1 dysregulation during decidualization may present a new perspective on infertility-related diseases.

**Supplementary Information:**

The online version contains supplementary material available at 10.1186/s12958-024-01188-9.

## Background

Human endometrium tissue is highly dynamic going through proliferative, secretory, and menses phases during a regular menstrual cycle [1–3]. Correspondingly, its functional layer exhibits steroid hormone-dependent proliferation, progesterone-stimulated differentiation, and shedding in the absence of the trophoblast [3]. After the postovulatory phase, the rising circulating levels of progesterone drive human endometrial stromal cells (HESCs) to differentiate into decidual cells, which is referred to as the decidualization process [2–5]. Decidualization is the morphological transformation of HESCs from a proliferating fibroblastic phenotype to an enlarged and rounded epithelial shape, accompanied by secretion of prolactin (PRL) and insulin-like growth factor binding protein-1 (IGFBP-1), which is required for female fertility [2, 3, 5]. In the presence of a trophoblast, the decidualized endometrium will be maintained through the increased level of progesterone. Otherwise, it will be shed away with a rapid drop of the progesterone level [3]. A successful decidualization process is an essential prerequisite for embryo implantation and subsequent placenta formation.

During decidualization, progesterone (P4) classically affects the endometrium through activation of two major well-characterized progesterone receptor PR-A and PR-B [5]. Progesterone receptor membrane component 1 (PGRMC1), one of the non-classical progesterone receptors, also rapidly respond to progesterone during decidualization; however, its function in this process is still being elucidated. In the human endometrium, PGRMC1 is abundantly expressed during the proliferative phase of the menstrual cycle in both endometrial and stromal cells. Whereas, in the secretory phase its expression levels dramatically decreased [6]. Overexpression of PGRMC1 in primary HESCs abrogated decidualization [7] and reduced PGRMC1 expression observed in multiple gynecological and obstetrics diseases [8–10]. Therefore, PGRMC1 was proposed as a fertility stabilizer to decidualization, whose expression must be finely tuned during the entire decidualization to support female fertility [11]. How this is achieved remains an enigma.

The prohibitin proteins (PHBs), prohibitin-1 (PHB1) and prohibitin-2 (PHB2), are ubiquitously expressed and highly conserved in eukaryotic cells [12]. PHBs has been reported to act as transcriptional corepressors for ERα in vitro and in vivo [13–15]. Loss of PHBs led to dysfunctional mitochondria, further resulting in male infertility and ovarian aging in females [16, 17]. Besides, PHB1 is downregulated in the eutopic and ectopic endometrium of patients with endometriosis compared to women without endometriosis [18]. An uterus-selective, conditional PHB2 knockout mouse model showed a subfertility phenotype with litters reduced both in number and size [19]. This implies that appropriate protein levels of PHB1/2 as well as of PGRMC1 are required for optimal uterine function and fertility. In breast cancer cells, progestin-activated PGRMC1 associated with PHBs to stimulate cellular proliferation [20]. Binding of activated PGRMC1 to PHBs was accompanied by decreased PHBs-ERα-interaction, resulting in elevated expression of ER-dependent genes. Whether the progestin-depended interaction between PHBs and PGRMC1 also occurs during decidualization has never been characterized before. Therefore, the role of their interaction with regards to female fertility remains to be elucidated.

In this study, we aimed to explore the functional role of PGRMC1 and PHBs, and their interplay for successful decidualization.

## Materials and Methods

### Data sources

We collected the associated gene expression profiles in publicly available Gene Expression Omnibus (GEO) database (https://www.ncbi.nlm.nih.gov/geo/). Samples from different menstruation phases (proliferative/PE, early secretory/ESE, mid-secretory/MSE, late secretory/LES) were chosen from GSE4888 and GSE56364 to detect expression of PGRMC1 [21, 22]. All raw data were background-subtracted and normalized.

### Cell culture

The hTERT-immortalized human endometrial stromal cells (T-HESCs) were purchased from abm (T0533). Both the cell lines T-HESCs and St-T1 were maintained in phenol-red free Dulbecco’s Modified Eagle Medium//Ham’s F12 (DMEM/F12; Gibco, Thermo Fisher Scientific, 11039021) medium supplemented with 10% (*v/v*) charcoal-stripped fetal bovine serum (Thermo Fisher Scientific, 12676029), 100 units/mL penicillin–streptomycin (Thermo Fisher Scientific, 2321118), 50 µg/ml gentamycin sulfate (Biowest, L0012), 200 µM sodium pyruvate (Biowest, L0624) and 1.5 g/L sodium bicarbonate (Biowest, L0680) (hereafter referred to as complete medium) in a humidified incubator at 37 °C in the presence of 5% CO_2_. Cells (passage number < 10) were regularly tested negative for mycoplasma.

### Chemical compounds

AG205 (Sigma-Aldrich) was diluted in 2% charcoal-stripped FBS complete medium to 15 mM. Medroxyprogesterone acetate (MPA) and 8-Br-cAMP MPA (cAMP) were prepared from a 10 mM and 5 mM stock solution, respectively.

### MTT Assay

We measured activated cellular metabolism as a surrogate for proliferation by performing the MTT assay. Briefly, T-HESCs cells (5 X 10^3^ cells per well) were seeded in triplicates in 96-well plate in complete medium and grown for 24 h. After the attachment, cells were either treated with or without induction cocktail in decidualization medium. On the day of assay, cells were incubated with 0.25 mg/ml MTT (Sigma-Aldrich) in decidualization medium for 3 h at 37 oC. Following 1 h of incubation with DMSO at 37 oC and 300 rpm in a microplate shaker, absorption was measured at 540 nm using TECAN Spark® spectrophotometer.

### Immunofluorescence staining

Cells were seeded and cultured in chamber slides (Nunc Lab-Tek, Thermo Fisher Scientific C7182-1PAK) fixed with 4% formaldehyde (Sigma-Aldrich, 20649296018) for 10 min at room temperature (RT), washed with washing buffer (Dako, Glostrup, Denmark, S3006) (3 × 5 min each). Then, cells were permeabilized with 0.1% Triton X-100 (Sigma-Aldrich, T8787) in PBS for 10 min at RT and washed with washing buffer again (3 × 5 min each). DAKO protein block buffer (Dako, X0909) was added and incubated for 1 h at RT before incubating with primary antibodies specific for PGRMC1 (Abcam, ab48012), PHB1 (Abcam, ab75766), PHB2 (Cell signaling, 14084S) and Vimentin (Abcam, ab02547) overnight at 4 °C. The next day, cells were washed with washing buffer (3 × 5 min each) and incubated with secondary antibodies (Donkey-anti-goat, Alexa 488: Invitrogen, A11055; Donkey-anti-rabbit, Alexa 488: Invitrogen, A31573) for 1 h at RT in a humidified chamber in the dark. Nucleic acid was stained with DAPI (Thermo Fisher Scientific, 15733122) simultaneously with co-incubated secondary antibodies. After the final wash, the cells were mounted with Fluorescent Mounting Medium (Dako, S3023). Negative controls were prepared for each sample following the same staining procedure with isotype controls instead of primary antibodies. Fluorescence signals were detected with an Axioplan 2 Imaging fluorescence microscope (Carl Zeiss Microscopy GmbH, Jena, Germany).

### Proximity ligation assay

The *in-situ* proximity ligation assay (PLA) procedure was performed with the Duolink® PLA Kit (Sigma-Aldrich, DUO92008) and following the manufacturers protocol. The cells were incubated with the primary antibodies i.e., anti-PGRMC1 (Abcam, ab48012) with PHB1 (Abcam, ab75766) and PHB2 (Cell signaling, 14085S) overnight at 4 °C. The slides were washed twice for 5 min with buffer A, followed by incubation with the PLA probes (anti-goat PLUS and anti-rabbit MINUS) in antibody diluent for 60 min at 37 °C. After washing twice for 5 min with buffer A, ligation was performed using ligase diluted in ligation buffer for 30 min at 37 °C. Then the cells were washed with buffer A before incubation for 100 min with amplification stock solution at 37 °C. After washing twice for 10 min with buffer B, nuclear DNA was labeled with DAPI for 10 min and slides were mounted with mounting medium. Negative PLA control was performed using respective isotype control antibodies (isotype goat, Abcam, ab37373; isotype rabbit, Abcam, ab37415). Red fluorescence dots inside the cellular areas representing a single protein–protein interaction were quantified using image J software.

### Western blotting

Cell suspensions were washed twice with ice cold PBS (Thermo Fisher Scientific, 2176323) and lysed in RIPA lysis buffer (50 mM TRIS (Sigma-Aldrich, 74,385), 150 mM NaCl (VWR corporation, 16C030032), 1% NP-40 (Sigma-Aldrich, 74,385), 0.5% Sodium deoxycholate (Sigma-Aldrich, D6750), 0.1% SDS (Sigma-Aldrich, S34121136), containing protease inhibitor (Roche, 49,422,800) and phosphatase inhibitor (Roche, 49121300). Protein concentration was determined using Pierce™ BCA Protein Assay Kit (Thermo Fisher Scientific, 23225). An amount of 20 µg of total protein was supplemented with 4 × Laemmli buffer (Bio-Rad, Feldkirchen, Germany, 1610747) containing 2-Mercaptoethanol (Sigma-Aldrich, M6250) and loaded onto Mini-PROTEAN® Precast Gels (Bio-Rad, 4568123) and separated via SDS-PAGE in SDS buffer (25 mM TRIS, 192 mM glycine (Sigma-Aldrich, 50046), 0.1% SDS, pH 8.3) at 100-150 V. Protein was transferred to Immun-Blot® PVDF Membranes (Bio-Rad,1620177) overnight at 4 °C and 10 mA in blotting buffer (20 mM TRIS, 200 mM glycine, 20% (v/v) methanol). Unspecific binding was blocked by incubation of the PVDF membrane with 5% skim milk powder (Sigma-Aldrich, 70166) in TRIS-buffer (20 mM TRIS, 150 mM NaCl, pH 7.6) containing 0.1% Tween 20 (TBS-T) for 1 h at RT. Primary antibodies including PGRMC1 (Cell signaling, Danvers, MA, USA, D6M5M), PHB1 (Cell signaling, 2426S), PHB2 (Cell signaling, 14085S) and ß-actin (Santa Cruz Biotechnology, sc-4778) were added in 5% skim milk—TBS-T and incubated overnight at 4 oC. Secondary antibodies were applied in 5% skim milk—TBS-T at RT for 1 h. Proteins were detected using Amersham™ ECL™ Western Blotting Detection Reagent (Cytiva, 17190731).

### Subcellular protein fractionation

A subcellular protein fractionation kit (Thermo Fisher Scientific) was used to fractionate proteins into cytoplasmic, membrane, and nuclear fractions. Cells were harvested as pellets. The pellet was lysed with cytoplasmic extraction buffer, membrane extraction buffer, and nuclear extraction buffer. Primary antibodies specific for β-actin (Santa Cruz Biotechnology), Calreticulin (Santa Cruz Biotechnology), and Histon H3 (Cell signaling) were used to indicate the purity of the cytoplasmic, membrane, and nuclear fractions, respectively.

### Co-immunoprecipitation

Co-immunoprecipitation was performed using the Pierce Co-IP kit (Thermo Fisher Scientific). Briefly, the anti-PGRMC1 antibody (Cell signaling) was first immobilized for 2 h using AminoLink Plus coupling resin. In parallel, cell pellets were resuspended in ice-cold IP Lysis buffer. An amount of 500 µg protein was incubated with resin at 4 °C overnight. After incubation, the resin was washed, and protein complexes bound to the antibody were eluted using elution buffer. Subsequent western blot analyses were performed as described before.

### Gene silencing (siRNA Transfection)

To knock down PGRMC1 expression in T-HESCs, FlexiTube GeneSolution (Qiagen) was used, containing four siRNA(s) that specifically target human PGRMC1 mRNAs. Cells were transfected with the final concentration of 10 nM *PGRMC1* siRNA(s) or negative control siRNA (siCTL) (Thermo Fisher Scientific) using Lipofectamine RNAiMAX Transfection Reagent (Thermo Fisher Scientific) according to recommended procedures. Afterwards, cells were treated with decidualization medium containing either induction cocktail or DMSO, and harvested at different time points for downstream experiments. For PHB1 and PHB2 mRNA expression inhibition (siPHB1, siPHB2: Qiagen), the same siRNAs concentration was used.

### Quantitative reverse-transcription PCR (qRT-PCR)

RNA was isolated using the RNeasy Mini Kit (Qiagen) according to the manufacturer’s specifications. Reverse transcription of RNA into cDNA was performed with the Omniscript RT kit (Qiagen) according to the manufacturer’s instructions. Quantitative PCR was performed using QuantiFast SYBR Green PCR Kit (Qiagen) and LightCycler ®480 System (Roche). Primers for *PGRMC1* (Qiagen), *PRL* (Qiagen) and *HPRT1* (Qrigene, Rockville, MD, USA). The delta-delta cycle threshold method was used to normalized expression to the reference gene HPRT1 [23, 24].

### Statistical analysis

A two-tailed paired Student’s *t*-test was used to analyze experiments comparing two experimental groups or two-way ANOVA for multiple comparisons of more than two groups. A value of *p* < 0.05 was considered significant. All statistical analyses were performed with GraphPad Prism 9.0. Results were reported as means with standard deviation.

## Results

### PGRMC1 expression profile during regular menstrual cycle

**Fig. 1:**
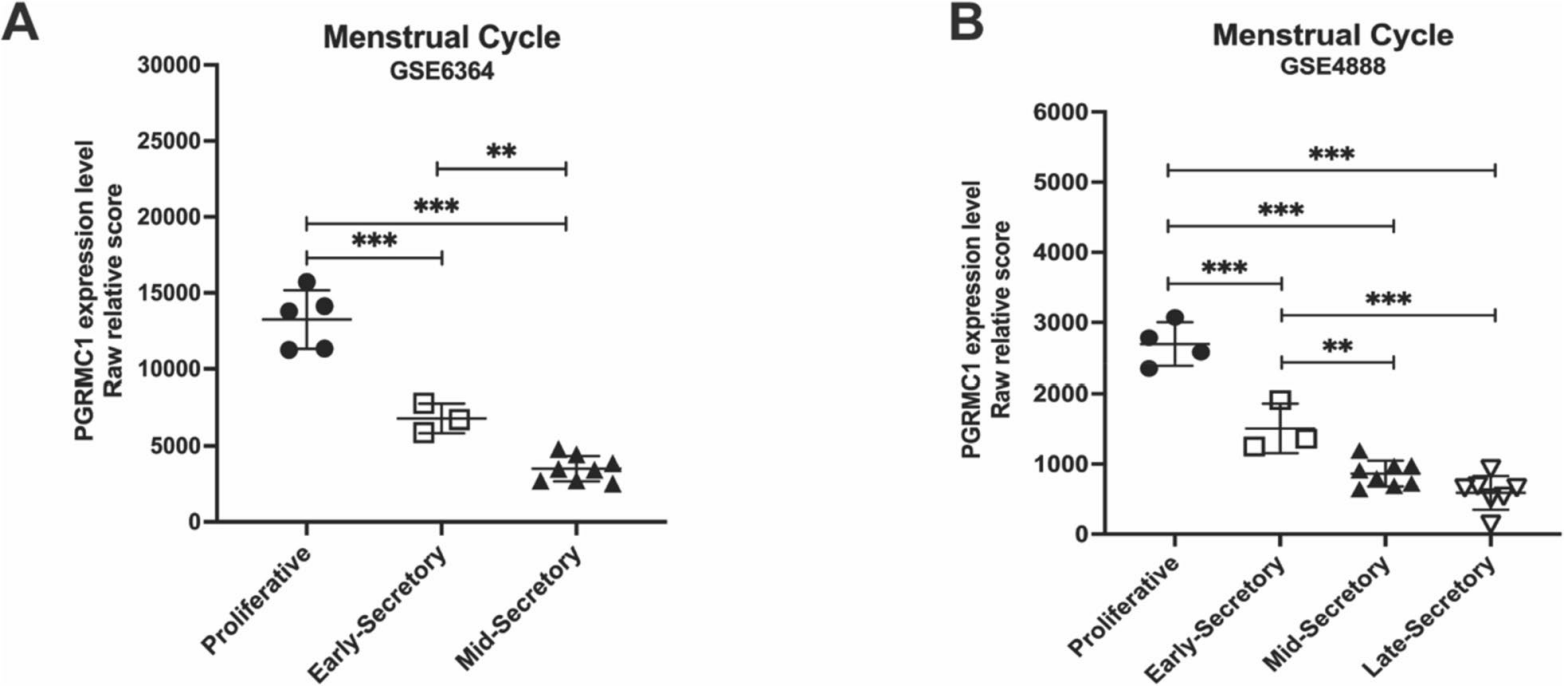
PGRMC1 expression profile during menstrual cycle. Relative transcript scores of PGRMC1 expression in different stages of a regular menstrual cycle (GSE6364) **(A)** and (GSE4888) **(B)**. Relative transcript scores of PGRMC1 expression level are shown as mean ± SEM. Statistical analysis was performed by two-way ANOVA. *p < 0.05, **p < 0.01, ***p < 0.001, ****p < 0.0001

To understand the dynamics of PGRMC1’s expression changes during normal decidualization, we initially investigated its expressional profile by mining publicly available RNA-sequencing data sets from endometrial biopsies (GEO accession numbers: GSE6364 and GSE4888). In a regular menstrual period, PGRMC1 mRNA level gradually decreased from the proliferative phase to the secretory (including early-, mid-, and late-) phase, manifesting the highest level in the proliferation phase and the lowest level in the late-secretory phase (Fig. 1A-B), consistent with previously reported data [9, 25]. This indicates that PGRMC1 may have an important role in regulating cellular proliferation and may not be required for decidualization in the secretory phase as it is consecutively decreased at mRNA level after progesterone stimulation. We hypothesized that the dynamic changes of PGRMC1 have an important role during the menstrual cycle that must be finely tuned.

### Rise-to-decline trend of PGRMC1 expression during in vitro decidualization

**Fig. 2:**
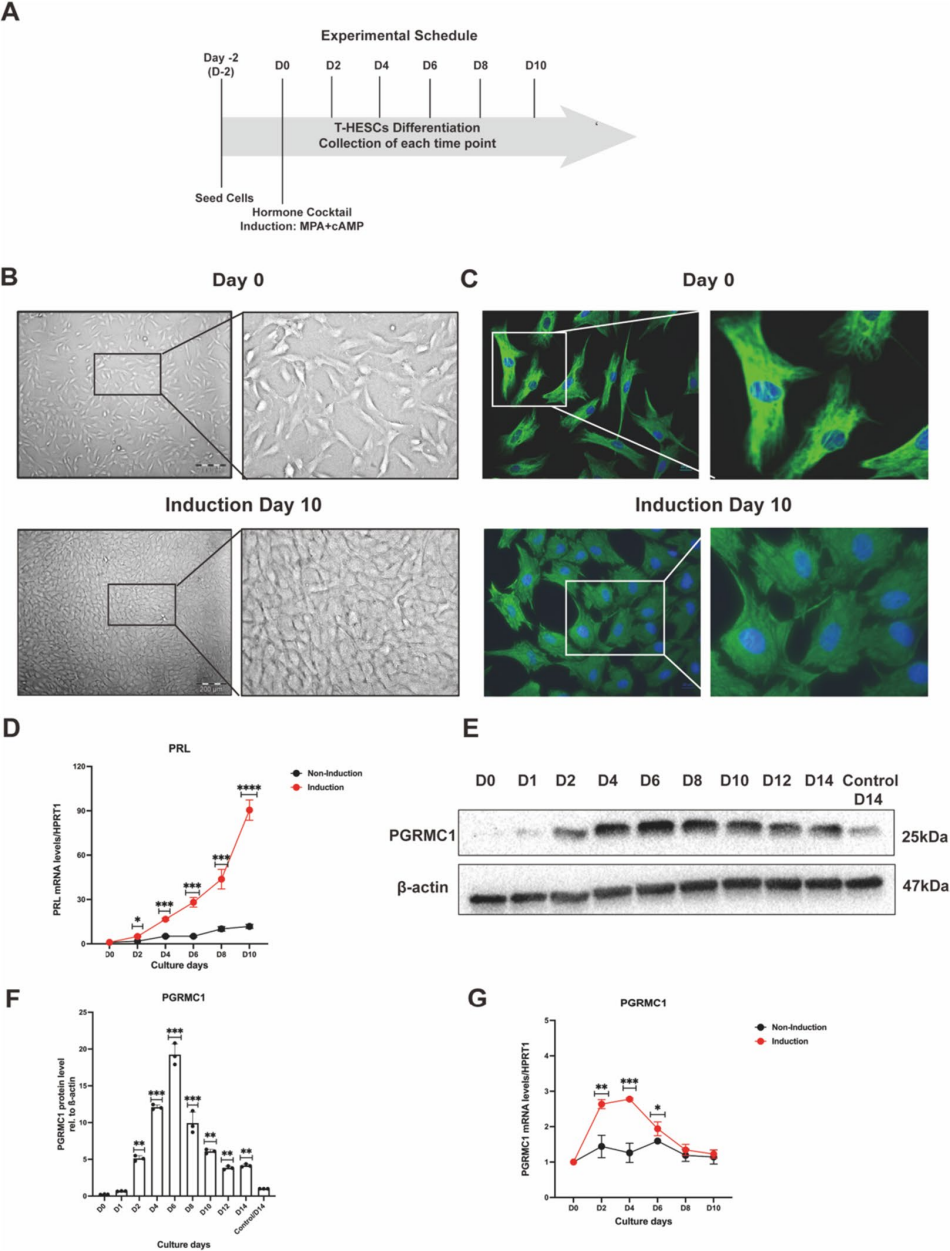
A rise-to-decline expression pattern of PGRMC1 was revealed by in vitro decidualization. **A** Schematic representation of in vitro decidualization system. The cellular morphology changes of T-HESCs on day 0 and day 10 were imaged with microscopy in bright filed **(B)** or immunofluorescence staining **(C).** PGRMC1 was stained by Alexa Fluor-488 (green), and the nucleus was stained by DAPI in blue. Scale bar: 200 µm. The mRNA expression levels of *PRL* in T-HESCs were analyzed with qRT-PCR when cells were cultured with MPA/cAMP (red line) for decidualization or DMSO (black line) as control **(D)**. The dynamic changes of PGRMC1 protein expression from 1 to 14 days induction and non-induction control (on Day 14) were measured by western blot **(E)** and the bar plot with the relative densitometric analysis of the corresponding PGRMC1 protein level (*p* value calculation based on ‘D0’) **(F)**. β-actin was used as a loading control. The mRNA expression levels of *PGRMC1* in T-HESCs during decidualization **(G)**. Results are shown as the mean ± SEM from three biological replicates. Statistical analysis was performed by two-way ANOVA. *p < 0.05, **p < 0.01, ***p < 0.001, ****p < 0.0001

To investigate our hypothesis, we established a hormone-inducible in vitro decidualization model in T-HESCs based on visualizing its morphological changes and by measuring the expression level of the decidual marker prolactin (PRL) (Fig. 2A). After being exposed to the decidualization induction cocktail consisting of the P4 analog MPA plus cAMP for 10 days, morphological changes of T-HESCs were inspected by microscopy in bright-field and by immunofluorescent analysis of the cytoskeletal marker vimentin. With this protocol, T-HESCs underwent a transformation from a fibroblast-like shape to a polygonal epithelial-like shape (Fig. 2B-C) accompanied with a significant increase of PRL mRNA expression compared to non-induced controls (Fig. 2D). Both the morphological changes and enhanced expression of PRL indicate a successfully established the hormone-induced decidualization model, allowing to investigate the role of PGRMC1 in decidualization.

We aimed to determine and modulate the expression level of PGRMC1 in our system to study its impact on the decidualization process. First, we determined the protein expression profile of PGRMC1 during the in vitro decidualization program. Intriguingly, we found that PGRMC1 expression gradually increased from the day the induction cocktail had been added (D0), peaking at day 6 (D6) post-induction, followed by a constant decrease until day 14 (D14) (Fig. 2E-F). This protein expression change could also be observed in the St-T1 cell line (Supplementary Fig. 1A). In contrast with the observation of gradually decreased mRNA levels of PGRMC1 during the secretory phase of the normal menstrual cycle (Fig. 1A-B), our in vitro decidualization model revealed that the promotion of cells into decidualized state comprises a PGRMC1 increasing phase and a PGRMC1 decreasing phase, both on protein and mRNA level (Fig. 2E-G). This data suggests that PGRMC1 was regulated at transcriptional level during decidualization. We termed this PGRMC1 protein dynamic changes as PGRMC1 ‘rise-to-decline’ changes in decidualization.

It is well known that increasing P4 levels initiate decidualization, although the activity of PGRMC1 in decidualization seems to be independent of P4 [3, 5]. Consistently, the T-HESC cells can go through the decidualization process treated with either P4, MPA, or cAMP (Supplementary Fig. 1B-C). Intriguingly, the PGRMC1 expression changes can be observed at each condition, which led us to the conclusion that the PGRMC1 rise-to-decline changes are a universal mechanism within the decidualization program.

### The rise-to-decline changes of PGRMC1 are required for decidualization

**Fig. 3:**
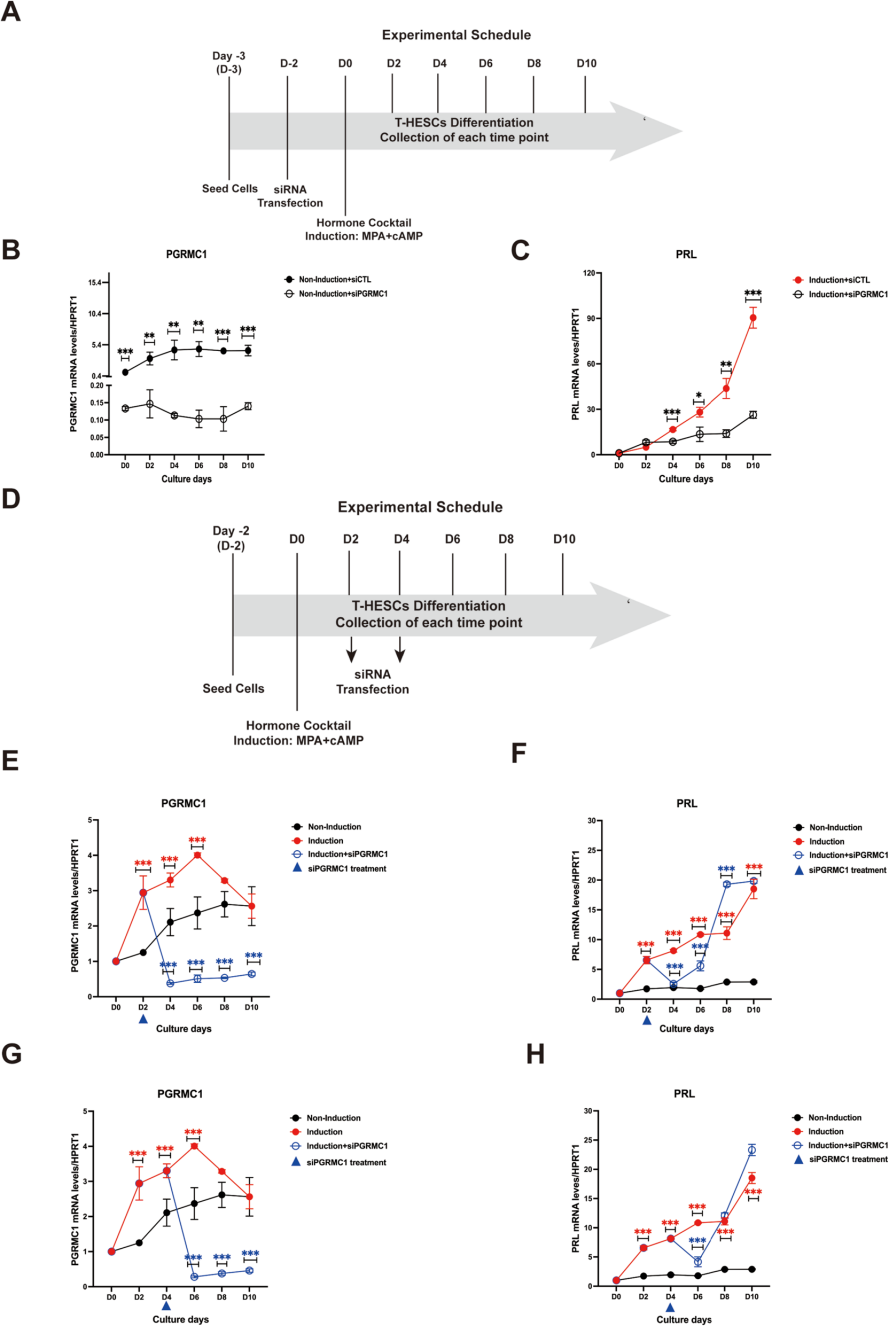
The rise-to-decline changes of PGRMC1 are required for decidualization. (**A**): Schematic representation of in vitro decidualization system after PGRMC1 downregulation by siRNA. qRT-PCR analysis of *PGRMC1* mRNA expression changes in T-HESCs transfected with either 10 nM of siRNA against PGRMC1 (siPGRMC1) or 10 nM control siRNA (siCTL) for up to 10 days **(B)**. The *PRL* mRNA expression level in T-HESCs after MPA/cAMP-induced decidualization upon transfection with 10 nM siPGRMC1 or siCTL, analyzed with qRT-PCR **(C)**. The workflow for PGRMC1 downregulation after decidualization induction **(D)**. mRNA expression levels of *PGRMC1* **(E, G)** and *PRL* **(F, H)** in T-HESCs treated with MPA/cAMP for decidualization induction (red line) and non-induction (black line). Blue lines indicate the mRNA levels of *PGRMC1* and *PRL* when transfected with 10 nM siPGRMC1 on the second (**E, F**) and fourth (**G, H**) day after decidualization induction, respectively. The statistical analysis of mRNA levels of *PGRMC1* (and *PRL*) between cells with non-induction and induction indicated by red stars, or cells with PGRMC1 knockdown after induction indicated by blue stars. Results are shown as the mean ± SEM from three independent biological replicates. Statistical analysis was performed by two-way ANOVA. *p < 0.05, **p < 0.01, ***p < 0.001, ****p < 0.0001

To explore the potential role of the PGRMC1 rise-to-decline changes during the decidualization, we firstly downregulated its expression before hormone induction with an optimized concentration of an siRNA-mix specific for PGRMC1 mRNA (Fig. 3A). Importantly, PGRMC1 mRNA levels were remained suppressed throughout 10 days post-siRNA-transfection (Fig. 3B). Likewise, expression of PGRMC1 protein was completely abrogated from day 2 (D2) to day 10 (D10) after siRNA transfection (Supplementary Fig. 2A-B).

T-HESCs with suppressed PGRMC1 expression were further treated with the decidualization induction cocktail. As indicated by the lack of morphological transformation and PRL production over 10 days of hormone treatment period (Fig. 3C, Supplementary Fig. 3), these cells did not undergo decidualization. Thus, in the absence of the progestin-induced PGRMC1 increasing phase decidualization failed.

These results prompted us to investigate if expression of PGRMC1 is needed for decidualization at the time point of induction – as a kind of a program switch – or later. To this aim, we postponed the transfection of PGRMC1 suppressing siRNAs to after the induction of decidualization. First, we treated T-HESC cells for 2 days with the combination of MPA and cAMP to induce decidualization followed siRNA transfection (Fig. 3D) and investigated the cells up to day 10 (D10) after induction. As expected, PGRMC1 mRNA levels started to decrease (Fig. 3E, blue line) after 2 days of transfection of PGRMC1-specific siRNAs and the PGRMC1 mRNA levels stayed below mRNA levels reached during normal induction of decidualization (Fig. 3E, red line). Interestingly, in addition to morphological changes (Supplementary Fig. 4), PRL mRNA expression level first dropped, but between D6 and D8 not only recovered to a comparative level to that of normal induction, was even four days earlier compared to the normal induction, indicating a promoted decidualization (Fig. 3F). We further measured the effects of knocking down PGRMC1 after 4 days of induction with MPA/cAMP on the decidualization program. The results are very similar to the outcome achieved when suppressing PGRMC1 after 2 days of induction (Fig. 3G-H). The results could be additionally reproduced in the St-T1 cell line (Supplementary Fig. 5A-D). Taken together, the PGRMC1 rise-to decline changes are required for a proper decidualization.

### PGRMC1-signal increases in the peri-nuclear region during decidualization

It has been reported that PGRMC1 translocates from cytoplasmic membranes to the nucleus during decidualization [7]. Recently, PGRMC1-mediated proteomic changes have been well characterized after decidualization, suggesting that PGRMC1 binds to proteins involved in translation, ATP generation, protein maturation, glucose transport, and lipid metabolism [26]. Almost all these proteins locate in the cytoplasm or on membranes, but not in the nucleus. This raises the question of why proteins interacting with PGRMC1 are barely found to be in the nucleus.

**Fig. 4:**
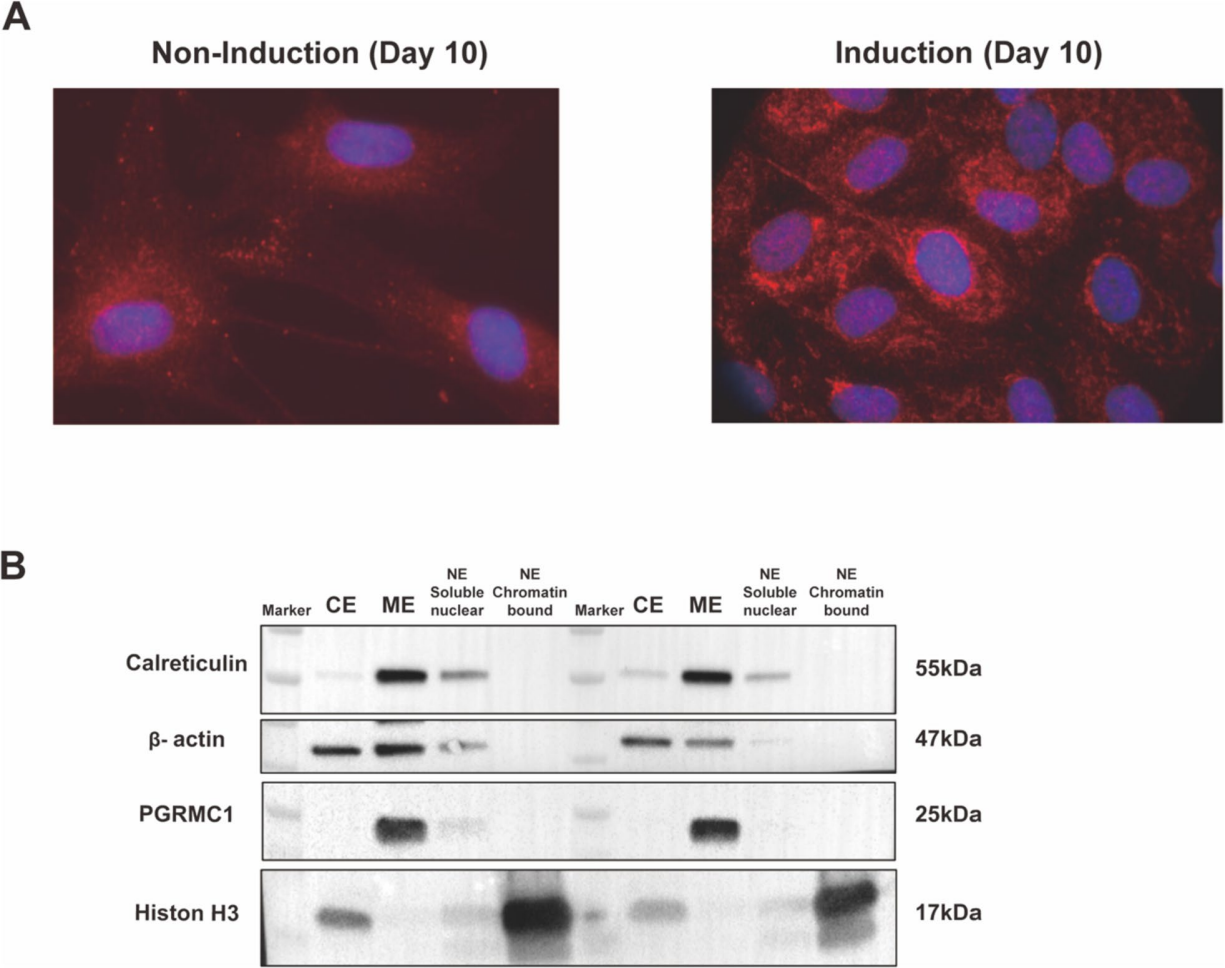
Prei-nuclear PGRMC1-signal increased during decidualization. (**A**): Immunofluorescence staining of PGRMC1 in T-HESCs treated with DMSO (left) as control or MPA/cAMP (right) for 10 days decidualization induction. PGRMC1 shows in red and the nucleus was stained with DAPI in blue. Scale bar: 200 µm. (**B**) Analysis of PGRMC1 localization by subcellular fractionation in T-HESCs treated with DMSO (left) or MPA/cAMP (right), measured by western blot. PGRMC1 was immunoblotted in equal amounts of cytoplasmic (CE), membrane (ME), and nuclear (NE) biomaterial. Compartment-specific markers: Calreticulin (55 kDa), β-actin (47 kDa), and Histon H3 (17 kDa) were used as loading controls for the membrane, cytoplasmic, and nuclear fractions, respectively

To better understand the question, we initially assessed PGRMC1 protein subcellular localization by immunofluorescence. Without induction of decidualization PGRMC1 was essentially located in the cytoplasm, but more intense signals were observed around the nucleus after 10 days induction (Fig. 4A). To further verify these observations, we fractionated the cells into soluble parts containing cytoplasm, membrane, and nucleus and detected the PGRMC1 protein by western blot. In line with the immunofluorescence results, PGRMC1 was only observed in the membrane fraction but not in the nucleus (Fig. 4B). This indicates an accumulation of PGRMC1 in the peri-nuclear region during hormone-induced decidualization.

### Interactions of PHB1/PHB2 to PGRMC1 mediate decidualization

**Fig. 5:**
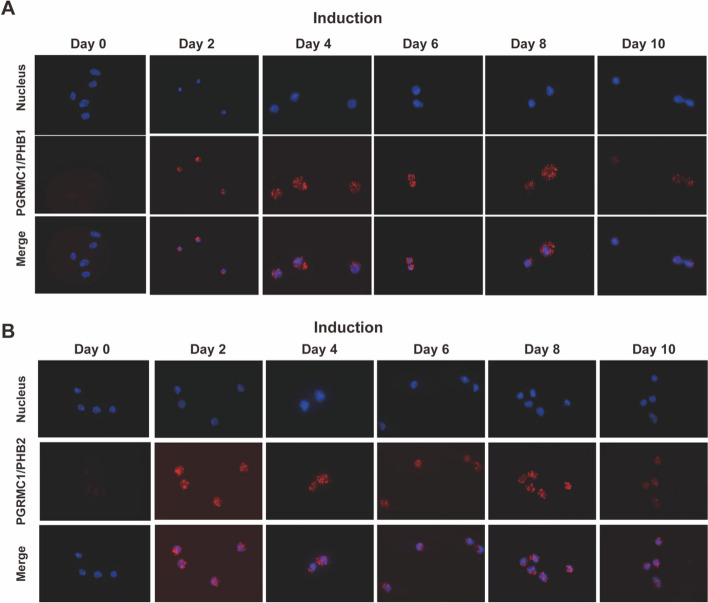
PGRMC1 interacts with PHBs during decidualization. The interactions between PGRMC1 and PHB1 (**A**) or PHB2 (**B**) in T-HESCs were analyzed with proximity ligation assay upon decidualization induction from day 2 to day 10. ‘Day 0’ indicates the induction day. Each red spot represents a single interaction. Nuclear stain: DAPI. Magnification 40 ×

We have recently demonstrated in breast cancer cells, that progestin-activated PGRMC1 interacts with PHB1/PHB2 resulting in enhanced ERα-dependent transcription and cell proliferation [27]. In analogy, here we found that PGRMC1 colocalized with PHB1 and PHB2 in the cytoplasm and at the nucleus periphery after induction, whereas barely colocalization signals could be observed without induction revealed by immunofluorescence (Supplementary Fig. 6A-B). This suggests a potential interaction between PHBs and PGRMC1 introduced by progestin treatment. Then, PLA was performed to further explore the associations between PGRMC1 and PHB1/2. Upon induction, a significantly higher PLA signal suggesting the interaction of PGRMC1 to PHB1/2 could be observed compared to the control (Fig. 5A-B, Supplementary Fig. 7A-B).

**Fig. 6:**
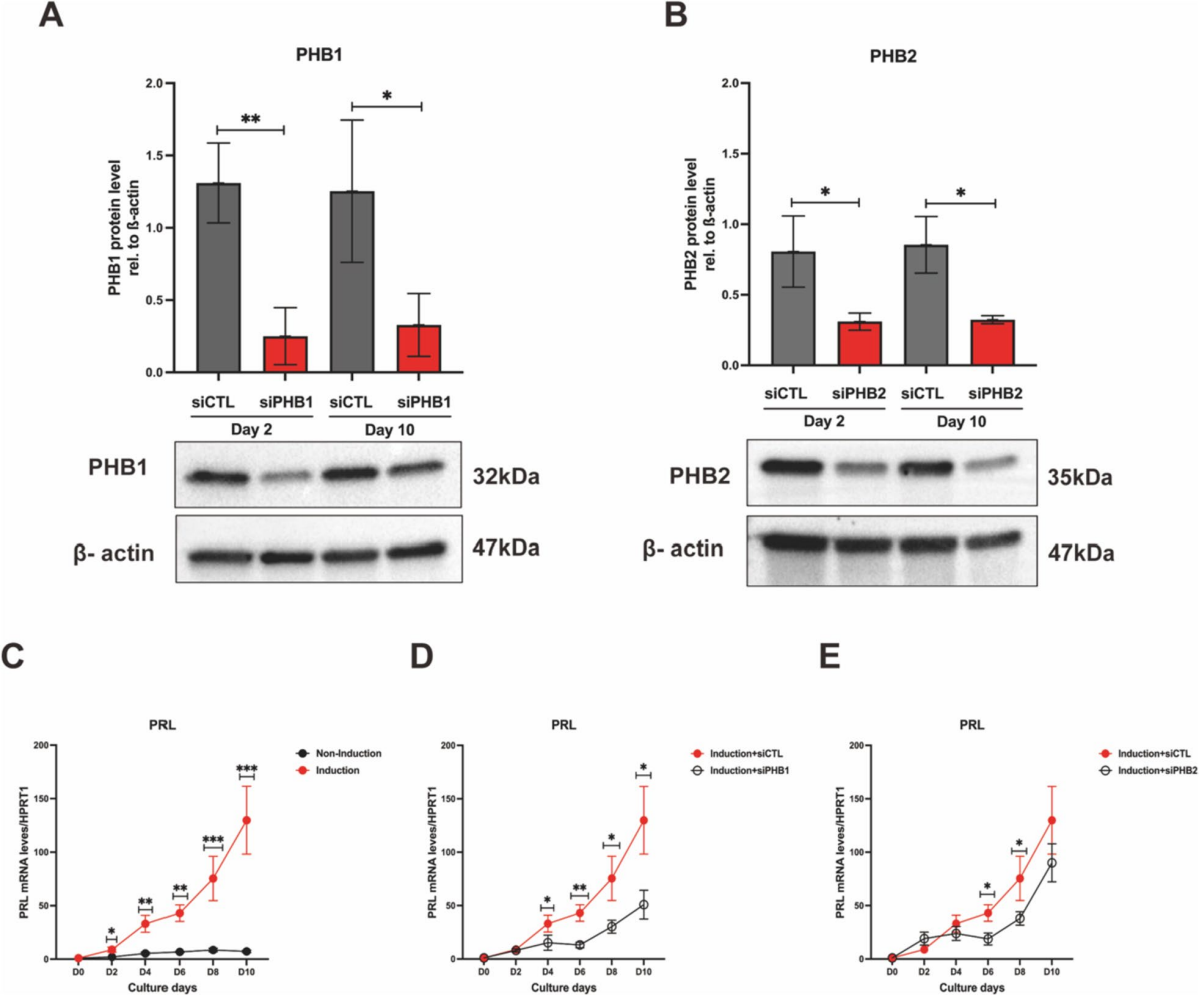
Downregulation of PHBs partly impairs decidualization. The PHBs protein expression level on day 2 or day 10 after transfection of T-HESCs with 10 nM siPHB1 (**A**), 10 nM siPHB2 (**B**), respectively, was analyzed by western blot. Densitometric analysis was performed with imagej and values were normalized to β-actin. The *PRL* mRNA expression changes in T-HESCs with (red line) and without (black line) induction was determined by qPCR and normalized to *HPRT1* as a reference gene (**C**). The *PRL* mRNA expression changes in T-HESCs transfected with 10 nM siPHB1 (**D**), 10 nM siPHB2 (**E**) upon decidualization induction were determined by qPCR. Results are shown as the mean ± SEM from three biological replicates. Statistical analysis was performed by two-way ANOVA. *p < 0.05, **p < 0.01, ***p < 0.001, ****p < 0.0001

To explore the function of PGRMC1-PHBs interaction during decidualization, we downregulated PHBs via siRNA transfection, reaching expression levels decreased by 60–80% compared to the control for individual PHBs (Fig. 6A-B). Knocking down each of the PHBs before hormone induction partly impaired the decidualization process (Fig. 6C-E), but the cells still could achieve morphological transformation (Supplementary Fig. 9). These effects on decidualization are comparable to the results achieved with suppressed PGRMC1.

### AG205 does not affect PGRMC1 rise-to-decline changes and decidualization

AG205 was reported to be a specific inhibitor of PGRMC1 and was broadly used to explore PGRMC1’s role in decidualization [28, 29]. Recent data, however, question the specificity of AG205 for PGRMC1 [29–31].

**Fig. 7:**
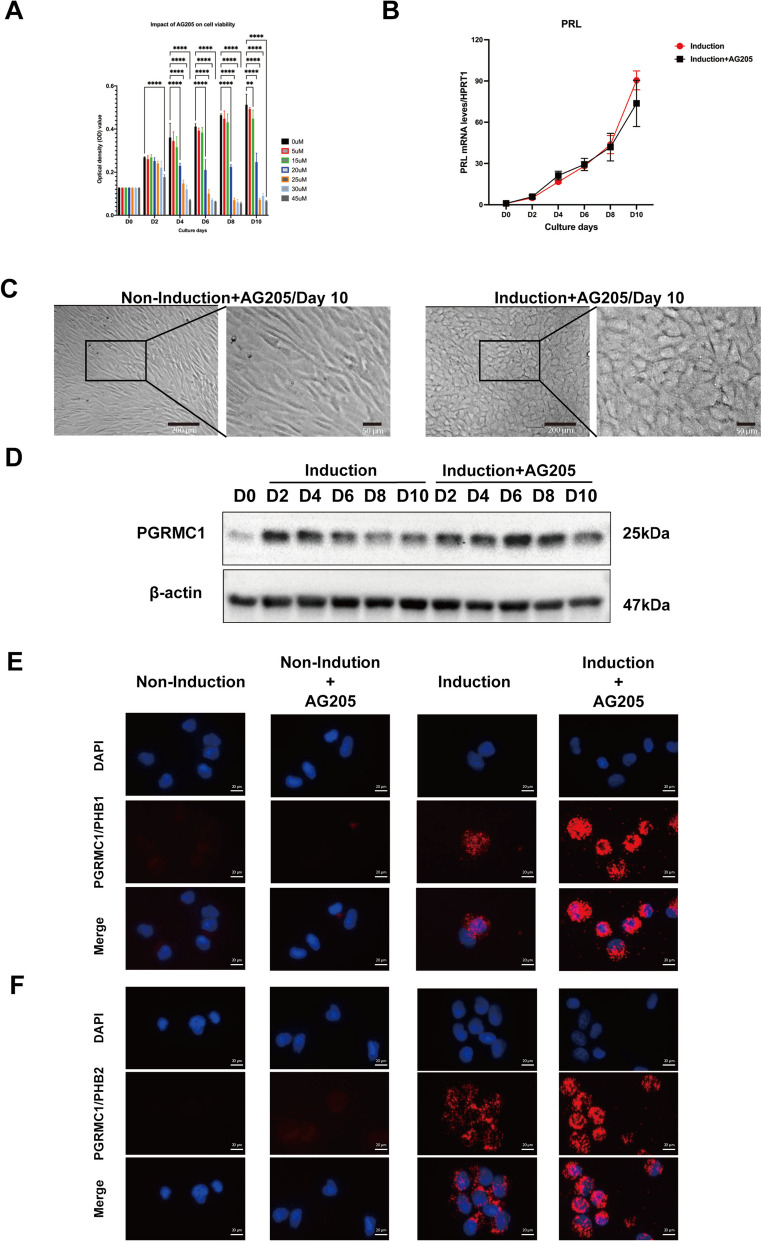
AG205 does not affect decidualization. The influence of AG205 on T-HESCs viability was performed after the cells were incubated with indicated concentrations of AG025 for 10 days and analyzed with colorimetric assay **(A)**. The absorbance values for cultures with AG205 were compared to the DMSO control (0 µM). The *PRL* mRNA expression levels were analyzed after cells were cultured with (black line) or without (red line) 15 µM AG205 **(B)**. Results are shown as the mean ± SEM from three independent biological replicates. Statistical analysis was performed by two-way ANOVA. *p < 0.05, **p < 0.01, ***p < 0.001, ****p < 0.0001. The cellular morphology changes of T-HESCs were imaged with microscopy in the bright field when cells were cultured without (upper panel) or with (down panel) MPA/cAMP upon 15 µM AG205 treatment **(C)**. Scale bar: 200 µm. The PGRMC1 protein expression changes in T-HESCs were analyzed by western blot when cells were treated with DMSO (left panel) or 15 µM AG205 (right panel) upon decidualization induction **(D)**. β-actin was used as a loading control. The interactions between PGRMC1 and PHB1 **(E)** or PHB2 **(F)** in T-HESCs were analyzed by proximity ligation assay when cells were cultured with 15 µM AG205 upon decidualization induction. Each red spot represents a single interaction. Nuclear stain: DAPI. Magnification 40 ×

Taking advantage of the critical role of PGRMC1 rise-to-decline changes for decidualization, we tested the effect of AG205 on PGRMC1 and the decidualization process. Since AG205 concentrations used in previous reports were high enough to impair cellular viability [30, 32, 33], we initially determined the appropriate concentration of AG205 that did not affect cell viability. In the MTT assay (Fig. 7A), a concentration below 15 µM had no (or a moderate) effect, whereas a concentration higher than 15 µM had a detrimental effect on cellular viability, which is consistent with previously reported [29]. In addition, decidualization was successfully achieved with T-HESCs treated AG205 with concentrations below 15 µM, as indicated by the increasing expression of PRL and the change in cell morphology (Fig. 7B-C). Furthermore, AG205 treatment did neither affect PCRMC1 protein level during decidualization, nor its rise-to-decline expression profile (Fig. 7D). Moreover, the interaction of PGRMC1 to PHBs was not disturbed as confirmed by PLA (Fig. 7E-F), which is in line with a previous report [32]. Based on these results, we propose that AG205 (< 15 µM) has no effect on the observed PGRMC1 functions during decidualization.

## Discussion

PGRMC1 has been demonstrated to play a role in various reproductive tissues, particularly endometrial stromal cells [27, 34–36]. It influences the decidualization process and female fertility [37]. We revealed that PGRMC1 protein exhibits a rise-to-decline pattern after progestin stimulation, essential for normal decidualization (Supplementary Fig. 10). Additionally, during decidualization, PGRMC1 interacted with PHB1 and PHB2, suggesting their joint contribution to the decidualization program. Despite the unclear mechanisms behind PGRMC1-dependent decidualization failure, PGRMC1 expression profile may serve as a useful fertility indicator.

Previous reports focused on PGRMC1 mRNA profile changes during decidualization, with few investigations into protein level dynamics. We measured both mRNA and protein levels of PGRMC1 after inducing decidualization and observed a rise-to-decline pattern. The observed increase and decrease of PGRMC1 protein expression fits into cyclic changes observed in vivo [25]. The overall dynamic changes of the PGRMC1 protein level during a normal menstrual cycle are composed of two peaks: one occurs in the secretory phase, as revealed in this study and the other one occurs in the proliferative phase as previously reported [9, 25]. It resembles estrogen dynamics during the menstrual cycle, suggesting PGRMC1 expression may be regulated by estrogen concentration or a similar mechanism [1–3]. As PGRMC1 overexpression in breast cancer cells leads to higher E2 secretion, T-HESCs E2 production might depend on PGRMC1 activation. Further research is needed to understand the relationship between estrogen and PGRMC1 expression, including the possibility of estrogen receptor-mediated transcription regulation.

Knocking down PGRMC1 before hormone treatment inhibited decidualization, highlighting its crucial role as a 'switch' at this stage. Appropriate PGRMC1 protein levels are needed to initiate decidualization upon P4/cAMP stimulation. The PGRMC1 rise-to-decline pattern can be induced by various treatments (Supplementary Fig. 1), suggesting a common signaling pathway that correlates with decidualization, which requires further investigation. PGRMC1 seems less necessary after decidualization initiation, as knocking it down either does not affect or even facilitates the process. It is unclear why PRL expression initially drops and then increases when PGRMC1 is knocked down after decidualization induction. Downregulating PGRMC1 after progestin treatment doesn't hamper decidualization, indicating its critical role during the increasing phase and induction. This aligns with observations that PGRMC1 downregulation in the secretory phase promotes decidualization [28]. Overall, PGRMC1 activation by P4 may facilitate the switch from cellular proliferation to decidualization initiation through various biological processes, while the mechanism of how downregulated PGRMC1 promotes decidualization warrants further investigation.

PGRMC1 has been known to occupy multiple subcellular locations, from endoplasmic reticulum, cytoplasm, plasma membrane, nucleus, and mitochondria, and its localization is regulated by including phosphorylation, ubiquitination, and sumoylation [34, 36]. In our study, we noticed an induction-associated peri-nuclear phenomenon, presenting as more intense signals in the peri-nuclear region at 10 day’s induction (Fig. 4A). PGRMC1’s peri-nuclear expression has been observed in various cells, suggesting its involvement in processes near or within the nucleus [7, 38, 39]. In our current study, we did not detect any nuclear PGRMC1 under the explored conditions, as demonstrated by subcellular fractionation analysis (Fig. 4B).

PGRMC1 associates with proteins involved in protein biosynthesis, intracellular transport, and mitochondrial activity to promote decidualization [26, 35]. However, little is known about how PGRMC1 interacts with these proteins to regulate decidualization. We found that PGRMC1 binds to PHBs at the nucleus periphery after P4 treatment, suggesting it may function as a scaffold protein for decidualization in endometriosis stromal cells. PGRMC1 could be anchored on the membrane of various organelles, co-transporting with them during decidualization-related morphological changes [40]. PHBs form a super complex in mitochondria, playing roles in lipid biogenesis, ATP generation, and more [12, 41]. Knocking down PHBs partially impaired decidualization, similar to PGRMC1 knockdown, suggesting PGRMC1-PHBs interactions may influence decidualization as a complex, requiring further investigation. We speculate that PGRMC1 binding to PHBs may inhibit cellular proliferation and facilitate differentiation, acting as a proliferation-differentiation switch.

We found that the small molecule AG205 neither affect PGRMC1-PHBs interaction, nor decidualization in our study (Fig. 7). Although AG205 has been shown to interact with PGRMC1 in vitro, its in vivo interaction remains unknown. Our data align with a recent study demonstrating that AG205 concentrations over 15 µM reduce cell proliferation, and concentrations above 30 µM result in cell death in HEC-1A and T-HESC cells [29]. Furthermore, our findings are consistent with a previous report indicating that a high concentration (50 µM) of AG205 did not affect decidualization [30].

This study on PGRMC1-PHB association, while informative, presents several limitations. Firstly, while PLA data suggests a close proximity and potential functional interaction between PGRMC1 and PHB, confirming a physical interaction necessitates additional in vivo interaction detection methods. Co-immunoprecipitation (Co-IP) has significant limitations, as it disrupts cellular integrity and loses crucial information about protein localization and physiologically relevant interactions due to cell lysis and potential interference from the buffer system, particularly for detecting weak, transient interactions or those confined to specific cellular compartments. Thus, in vivo crosslinking, which enables interaction detection in their native environment, should be considered. Secondly, the efficiency of PHB1 and PHB2 silencing via siRNA is lower compared to the almost complete knockdown of PGRMC1, suggesting the need for PHB knockout or stable knockdown models for clearer background results. Finally, a general limitation needs to be taken into account. The in vitro experiments performed in cell lines assured reproducibility within the established system, while primary patient endometrium tissues are highly heterogeneous and, in general, require analysis of a large cohort in order to obtain a statistically significant result. Consequently, this study primarily analyzed publicly available datasets and two cell lines. However, to validate the switch-like rise-to-decline expression pattern of PGRMC1 in vivo, future studies could utilize a mouse model with inducible PGRMC1-downregulation.

## Conclusion

Based on the results of our study, we postulate that P4/progestin-induced PGRMC1 rise-to-decline expression is essential to start the decidualization program, but, once decidualization started, PGRMC1 is not needed to drive it. Our PGRMC1-knockdown experiments demonstrated that PGRMC1 expression is specifically important at decidualization induction, leading to decidualization failure upon disruption. Taken together, we explained how dysregulated PGRMC1 expression could impact endometrial stromal cell decidualization, which may provide a new perspective on infertility-related diseases.

### Supplementary Information

**Additional file 1:** **Supplementary Fig. 1.** Rise-to-decline expression pattern of PGRMC1 is linked to the decidualization program. **(A)** PGRMC1 protein expression changes during 9 days of decidualization were measured by western blot in the St-T1 cell line. **(B)** PGRMC1 protein expression changes during 10 days of stimulation with MPA, cAMP, and DMSO, respectively, were measured by western blot in T-HESCs. **(C)** PGRMC1 protein expression levels on day 6 and day 10 when cultured with DMSO, nomegestrel (NOM), P4, cAMP, MPA/cAMP (M + A), and MPA, respectively, measured by western blot in T-HESCs. **Supplementary Fig. 2.** PGRMC1 is effectively downregulated by siRNA on protein level. **(A)** The PGRMC1 protein expression on day 2 and day 10 after transfection of T-HESCs with either 10 nM anti-PGRMC1 siRNA (siPGRMC1) or unspecific scrambled-control siRNA (siCTL). **(B)** A comparison of the PGRMC1 protein expression changes within 10 days after transfection of T-HESCs with either 10 nM siPGRMC1 or 10 nM siCTL. **Supplementary Fig. 3**. PGRMC1-downregulation before decidualization induction impairs morphological remodeling of T-HESCs. The cellular morphology changes of the T-HESCs induced with either DMSO (upper panel) or MPA/cAMP (down panel) after 10 days of siRNA treatment (siCTL, left panel; siPGRMC1, right panel). Scale bar: 200 µm. **Supplementary Fig. 4.** PGRMC1-downregulation after decidualization induction does not impair morphological remodeling of T-HESCs. The cellular morphology changes of the T-HESCs induced with either DMSO (non-induction, column 1) or MPA/cAMP (Induction, columns 2–4). I (column 3) and II (column 4) indicate that siRNA treatment on T-HESCs was conducted on day 2 or day 4 of decidualization induction, respectively. Scale bar: 200 µm. **Supplementary Fig. 5.** PGRMC1-downregulation after progestin induction does not impair decidualization in the St-T1 cell line. The mRNA expression levels of *PGRMC1* **(A, C)** and *PRL* **(B, D)** in St-T1 treated with MPA/cAMP for decidualization induction (red line), and non-induction (black line). The mRNA expression levels of *PGRMC1* and *PRL* in St-T1 cells transfected with 10 nM siPGRMC1 (blue line) on the second (**A, B**) and fourth day (**C, D**) after decidualization induction, respectively. Results are shown as the mean ± SEM from three independent biological replicates. Statistical analysis was performed by a two-way ANOVA test. *p < 0.05, **p < 0.01, ***p < 0.001, ****p < 0.0001. The red * indicates the comparison between the red and black lines. The blue * indicates the comparison between the red and blue lines. **Supplementary Fig. 6.** PGRMC1 and PHB1/PHB2 co-localize in T-HESCs. Double Immunofluorescence staining for PGRMC1 (red) and PHB1 (green) or PHB2 (green) in T-HESCs treated with DMSO **(A**) as control or MPA/cAMP **(B)** for decidualization induction. Magnification: 40x. Scale bar: 20 µm. **Supplementary Fig. 7.** PGRMC1 does not interact with PHBs without induction. The interactions between PGRMC1 and PHB1 **(A)** and PHB2 **(B)** in T-HESCs without induction were analyzed with proximity ligation assay from day 2 to day 10. Each red spot represents a single interaction. Nuclear stain: DAPI. Magnification 40X. **Supplementary Fig. 8.** PGRMC1 co-precipitate with PHB1/PHB2 upon decidualization induction. PGRMC1 was immunopurified from native whole cell lysates of T-HESCs using anti-PGRMC1 antibody. Western blot analyses of co-immunoprecipitated PHB1 (upper) and PHB2 (bottom) in T-HESCs with and without decidualization induction. **Supplementary Fig. 9.** Morphological changes during decidualization upon PHBs downregulation. The cellular morphology changes of the T-HESCs induced with either DMSO (non-Induction) or MPA/cAMP (Induction) upon either PHBs knockdown alone or both. Scale bar: 200 µm. **Supplementary Fig. 10.** Overview over the role of PGRMC1 in human endometrial decidualization. Upon stimulation with progesterone or MPA, the PGRMC1 rise-to-decline changes are essential for successful decidualization of the human endometrial cells (upper panel). With downregulated PGRMC1 expression before induction, the decidualization program cannot be carried out, leading to decidualization failure (bottom panel).

## Data Availability

The datasets analyzed in this study are available at Gene Expression Omnibus (GEO) database: https://www.ncbi.nlm.nih.gov/geo/ with the GEO accessions GSE6364 and GSE4888.
