# Supplementary figures and images for: Progesterone-induced progesterone receptor membrane component 1 rise-to-decline changes are essential for decidualization
Source: Reprod Biol Endocrinol. 2024 Feb 3;22:20. doi: 10.1186/s12958-024-01188-9 (PMC10837943; doi:10.1186/s12958-024-01188-9)

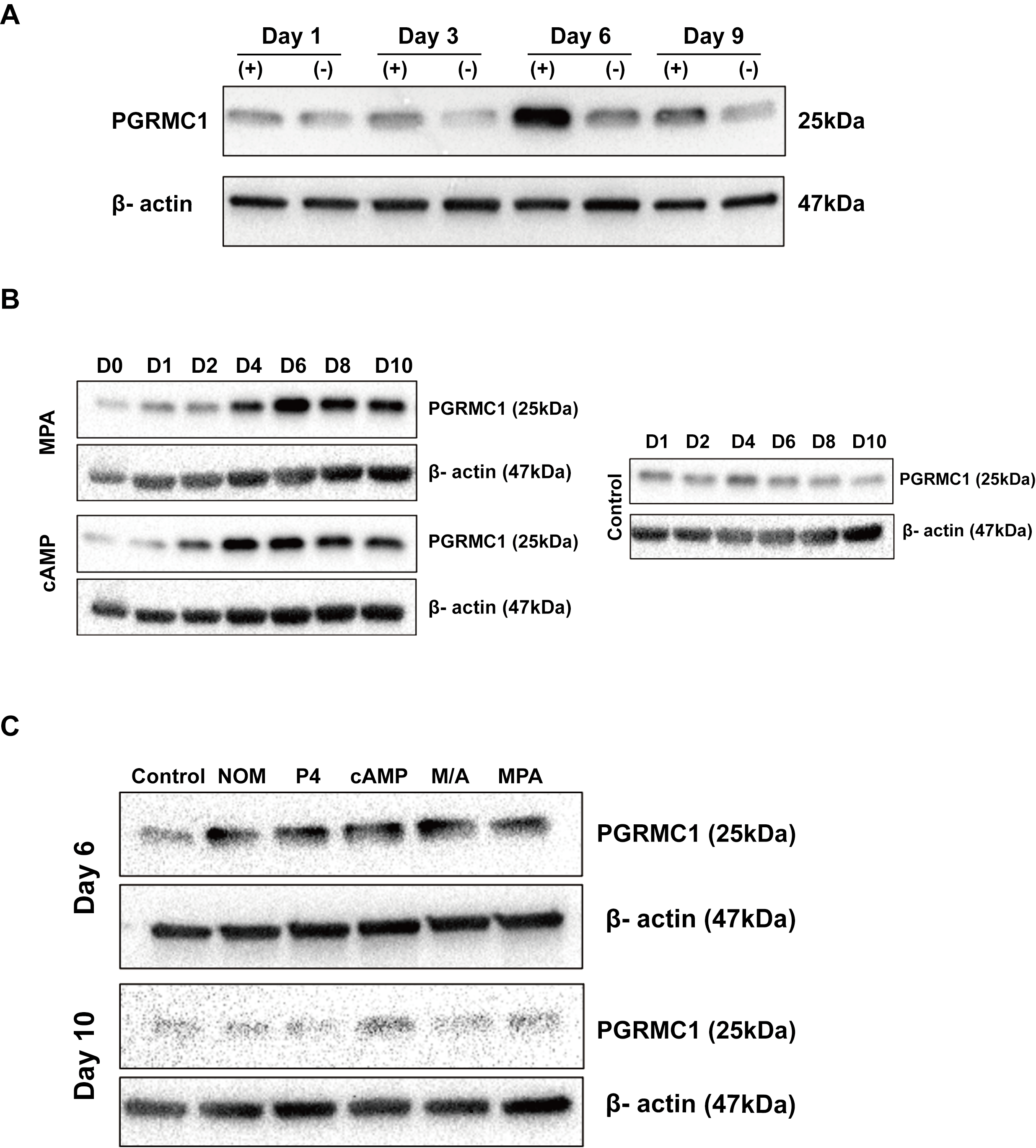

Supplement: Supplementary file 1 — Additional file 1: Supplementary Fig. 1. Rise-to-decline expression pattern of PGRMC1 is linked to the decidualization program. (A) PGRMC1 protein expression changes during 9 days of decidualization were measured by western blot in the St-T1 cell line. (B) PGRMC1 protein expression changes during 10 days of stimulation with MPA, cAMP, and DMSO, respectively, were measured by western blot in T-HESCs. (C) PGRMC1 protein expression levels on day 6 and day 10 when cultured with DMSO, nomegestrel (NOM), P4, cAMP, MPA/cAMP (M + A), and MPA, respectively, measured by western blot in T-HESCs. Supplementary Fig. 2. PGRMC1 is effectively downregulated by siRNA on protein level. (A) The PGRMC1 protein expression on day 2 and day 10 after transfection of T-HESCs with either 10 nM anti-PGRMC1 siRNA (siPGRMC1) or unspecific scrambled-control siRNA (siCTL). (B) A comparison of the PGRMC1 protein expression changes within 10 days after transfection of T-HESCs with either 10 nM siPGRMC1 or 10 nM siCTL. Supplementary Fig. 3. PGRMC1-downregulation before decidualization induction impairs morphological remodeling of T-HESCs. The cellular morphology changes of the T-HESCs induced with either DMSO (upper panel) or MPA/cAMP (down panel) after 10 days of siRNA treatment (siCTL, left panel; siPGRMC1, right panel). Scale bar: 200 µm. Supplementary Fig. 4. PGRMC1-downregulation after decidualization induction does not impair morphological remodeling of T-HESCs. The cellular morphology changes of the T-HESCs induced with either DMSO (non-induction, column 1) or MPA/cAMP (Induction, columns 2–4). I (column 3) and II (column 4) indicate that siRNA treatment on T-HESCs was conducted on day 2 or day 4 of decidualization induction, respectively. Scale bar: 200 µm. Supplementary Fig. 5. PGRMC1-downregulation after progestin induction does not impair decidualization in the St-T1 cell line. The mRNA expression levels of PGRMC1 (A, C) and PRL (B, D) in St-T1 treated with MPA/cAMP for decidualization in [file 12958_2024_1188_MOESM1_ESM.zip › Supplementary figure 1.tif]

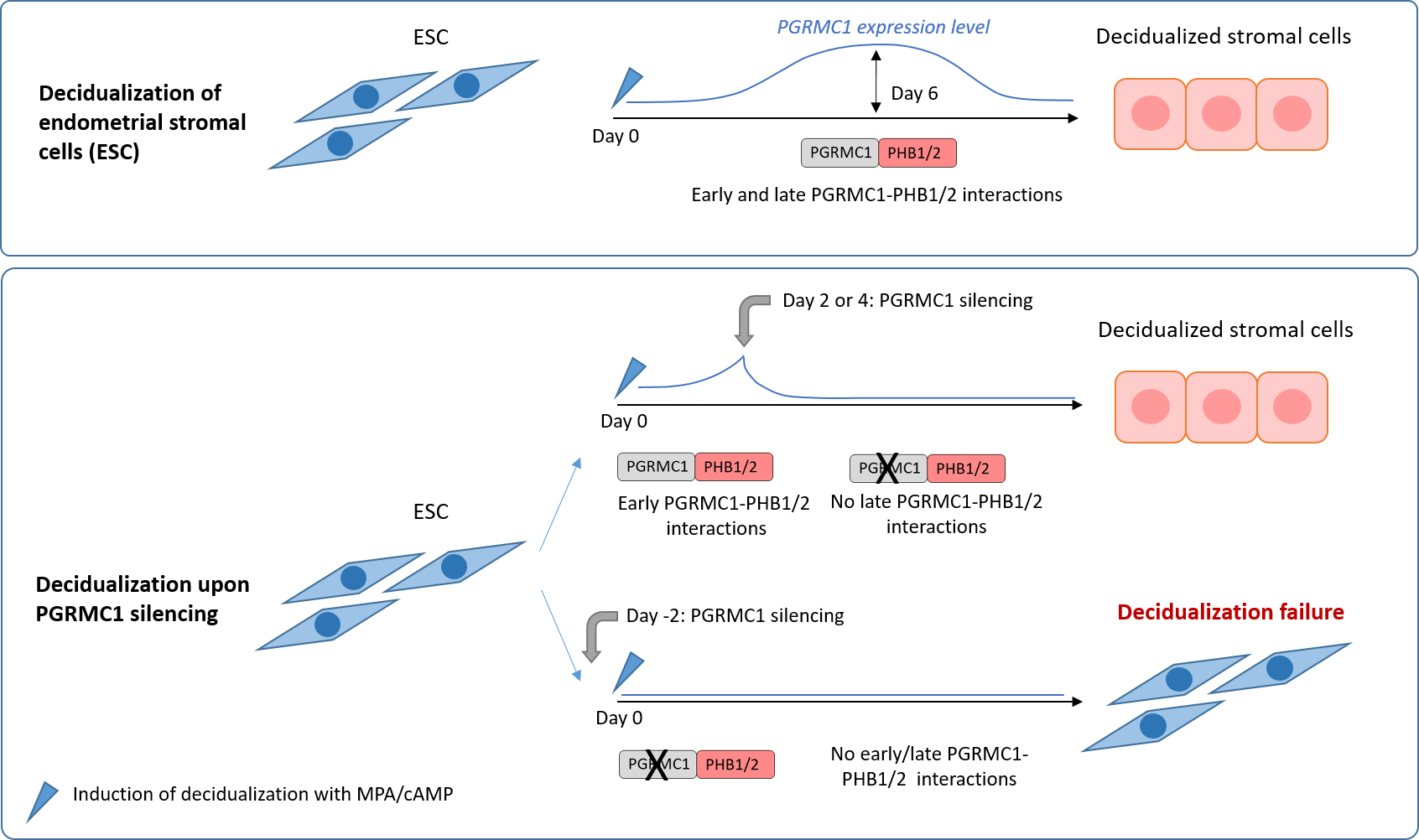

Supplement: Supplementary file 1 — Additional file 1: Supplementary Fig. 1. Rise-to-decline expression pattern of PGRMC1 is linked to the decidualization program. (A) PGRMC1 protein expression changes during 9 days of decidualization were measured by western blot in the St-T1 cell line. (B) PGRMC1 protein expression changes during 10 days of stimulation with MPA, cAMP, and DMSO, respectively, were measured by western blot in T-HESCs. (C) PGRMC1 protein expression levels on day 6 and day 10 when cultured with DMSO, nomegestrel (NOM), P4, cAMP, MPA/cAMP (M + A), and MPA, respectively, measured by western blot in T-HESCs. Supplementary Fig. 2. PGRMC1 is effectively downregulated by siRNA on protein level. (A) The PGRMC1 protein expression on day 2 and day 10 after transfection of T-HESCs with either 10 nM anti-PGRMC1 siRNA (siPGRMC1) or unspecific scrambled-control siRNA (siCTL). (B) A comparison of the PGRMC1 protein expression changes within 10 days after transfection of T-HESCs with either 10 nM siPGRMC1 or 10 nM siCTL. Supplementary Fig. 3. PGRMC1-downregulation before decidualization induction impairs morphological remodeling of T-HESCs. The cellular morphology changes of the T-HESCs induced with either DMSO (upper panel) or MPA/cAMP (down panel) after 10 days of siRNA treatment (siCTL, left panel; siPGRMC1, right panel). Scale bar: 200 µm. Supplementary Fig. 4. PGRMC1-downregulation after decidualization induction does not impair morphological remodeling of T-HESCs. The cellular morphology changes of the T-HESCs induced with either DMSO (non-induction, column 1) or MPA/cAMP (Induction, columns 2–4). I (column 3) and II (column 4) indicate that siRNA treatment on T-HESCs was conducted on day 2 or day 4 of decidualization induction, respectively. Scale bar: 200 µm. Supplementary Fig. 5. PGRMC1-downregulation after progestin induction does not impair decidualization in the St-T1 cell line. The mRNA expression levels of PGRMC1 (A, C) and PRL (B, D) in St-T1 treated with MPA/cAMP for decidualization in [file 12958_2024_1188_MOESM1_ESM.zip › Supplementary figure 10.tif]

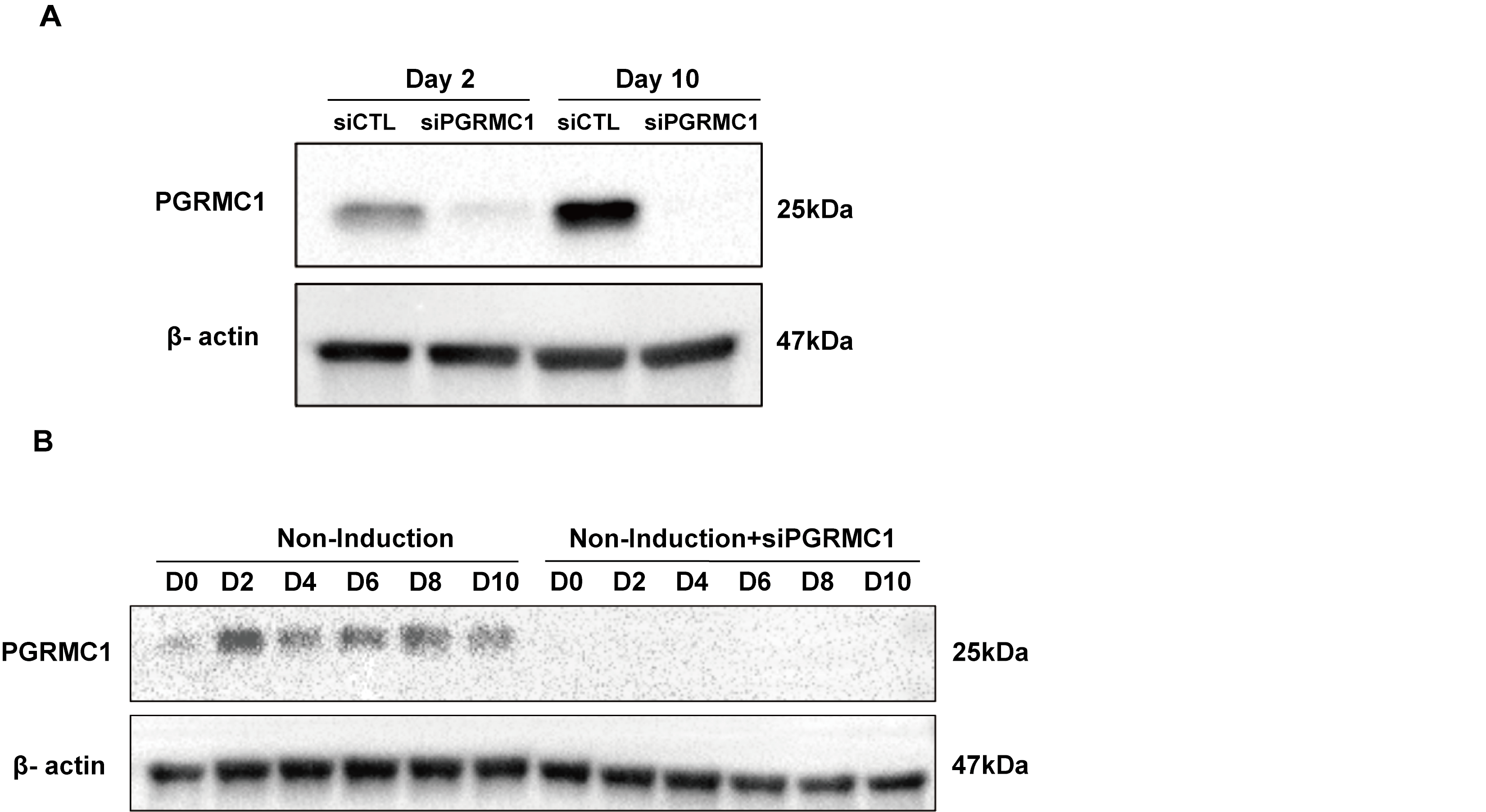

Supplement: Supplementary file 1 — Additional file 1: Supplementary Fig. 1. Rise-to-decline expression pattern of PGRMC1 is linked to the decidualization program. (A) PGRMC1 protein expression changes during 9 days of decidualization were measured by western blot in the St-T1 cell line. (B) PGRMC1 protein expression changes during 10 days of stimulation with MPA, cAMP, and DMSO, respectively, were measured by western blot in T-HESCs. (C) PGRMC1 protein expression levels on day 6 and day 10 when cultured with DMSO, nomegestrel (NOM), P4, cAMP, MPA/cAMP (M + A), and MPA, respectively, measured by western blot in T-HESCs. Supplementary Fig. 2. PGRMC1 is effectively downregulated by siRNA on protein level. (A) The PGRMC1 protein expression on day 2 and day 10 after transfection of T-HESCs with either 10 nM anti-PGRMC1 siRNA (siPGRMC1) or unspecific scrambled-control siRNA (siCTL). (B) A comparison of the PGRMC1 protein expression changes within 10 days after transfection of T-HESCs with either 10 nM siPGRMC1 or 10 nM siCTL. Supplementary Fig. 3. PGRMC1-downregulation before decidualization induction impairs morphological remodeling of T-HESCs. The cellular morphology changes of the T-HESCs induced with either DMSO (upper panel) or MPA/cAMP (down panel) after 10 days of siRNA treatment (siCTL, left panel; siPGRMC1, right panel). Scale bar: 200 µm. Supplementary Fig. 4. PGRMC1-downregulation after decidualization induction does not impair morphological remodeling of T-HESCs. The cellular morphology changes of the T-HESCs induced with either DMSO (non-induction, column 1) or MPA/cAMP (Induction, columns 2–4). I (column 3) and II (column 4) indicate that siRNA treatment on T-HESCs was conducted on day 2 or day 4 of decidualization induction, respectively. Scale bar: 200 µm. Supplementary Fig. 5. PGRMC1-downregulation after progestin induction does not impair decidualization in the St-T1 cell line. The mRNA expression levels of PGRMC1 (A, C) and PRL (B, D) in St-T1 treated with MPA/cAMP for decidualization in [file 12958_2024_1188_MOESM1_ESM.zip › Supplementary figure 2.tif]

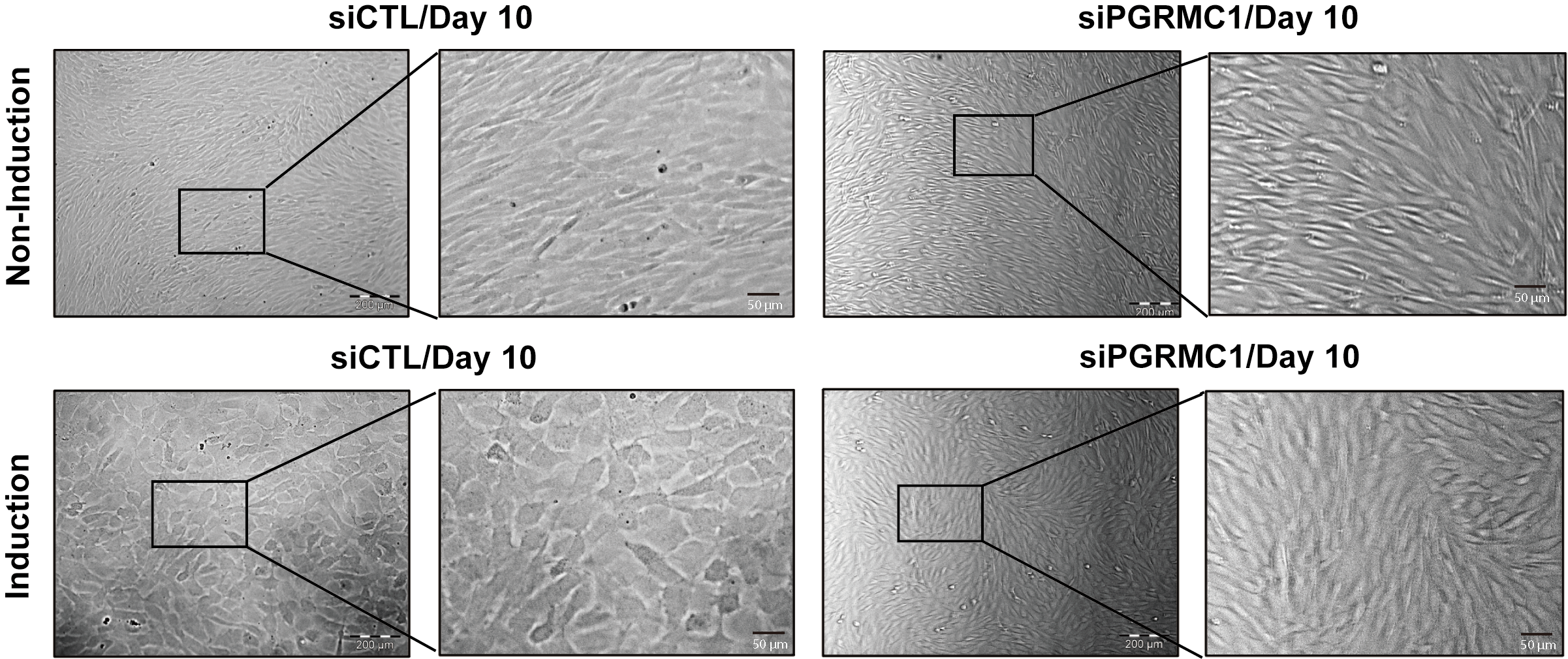

Supplement: Supplementary file 1 — Additional file 1: Supplementary Fig. 1. Rise-to-decline expression pattern of PGRMC1 is linked to the decidualization program. (A) PGRMC1 protein expression changes during 9 days of decidualization were measured by western blot in the St-T1 cell line. (B) PGRMC1 protein expression changes during 10 days of stimulation with MPA, cAMP, and DMSO, respectively, were measured by western blot in T-HESCs. (C) PGRMC1 protein expression levels on day 6 and day 10 when cultured with DMSO, nomegestrel (NOM), P4, cAMP, MPA/cAMP (M + A), and MPA, respectively, measured by western blot in T-HESCs. Supplementary Fig. 2. PGRMC1 is effectively downregulated by siRNA on protein level. (A) The PGRMC1 protein expression on day 2 and day 10 after transfection of T-HESCs with either 10 nM anti-PGRMC1 siRNA (siPGRMC1) or unspecific scrambled-control siRNA (siCTL). (B) A comparison of the PGRMC1 protein expression changes within 10 days after transfection of T-HESCs with either 10 nM siPGRMC1 or 10 nM siCTL. Supplementary Fig. 3. PGRMC1-downregulation before decidualization induction impairs morphological remodeling of T-HESCs. The cellular morphology changes of the T-HESCs induced with either DMSO (upper panel) or MPA/cAMP (down panel) after 10 days of siRNA treatment (siCTL, left panel; siPGRMC1, right panel). Scale bar: 200 µm. Supplementary Fig. 4. PGRMC1-downregulation after decidualization induction does not impair morphological remodeling of T-HESCs. The cellular morphology changes of the T-HESCs induced with either DMSO (non-induction, column 1) or MPA/cAMP (Induction, columns 2–4). I (column 3) and II (column 4) indicate that siRNA treatment on T-HESCs was conducted on day 2 or day 4 of decidualization induction, respectively. Scale bar: 200 µm. Supplementary Fig. 5. PGRMC1-downregulation after progestin induction does not impair decidualization in the St-T1 cell line. The mRNA expression levels of PGRMC1 (A, C) and PRL (B, D) in St-T1 treated with MPA/cAMP for decidualization in [file 12958_2024_1188_MOESM1_ESM.zip › Supplementary figure 3.tif]

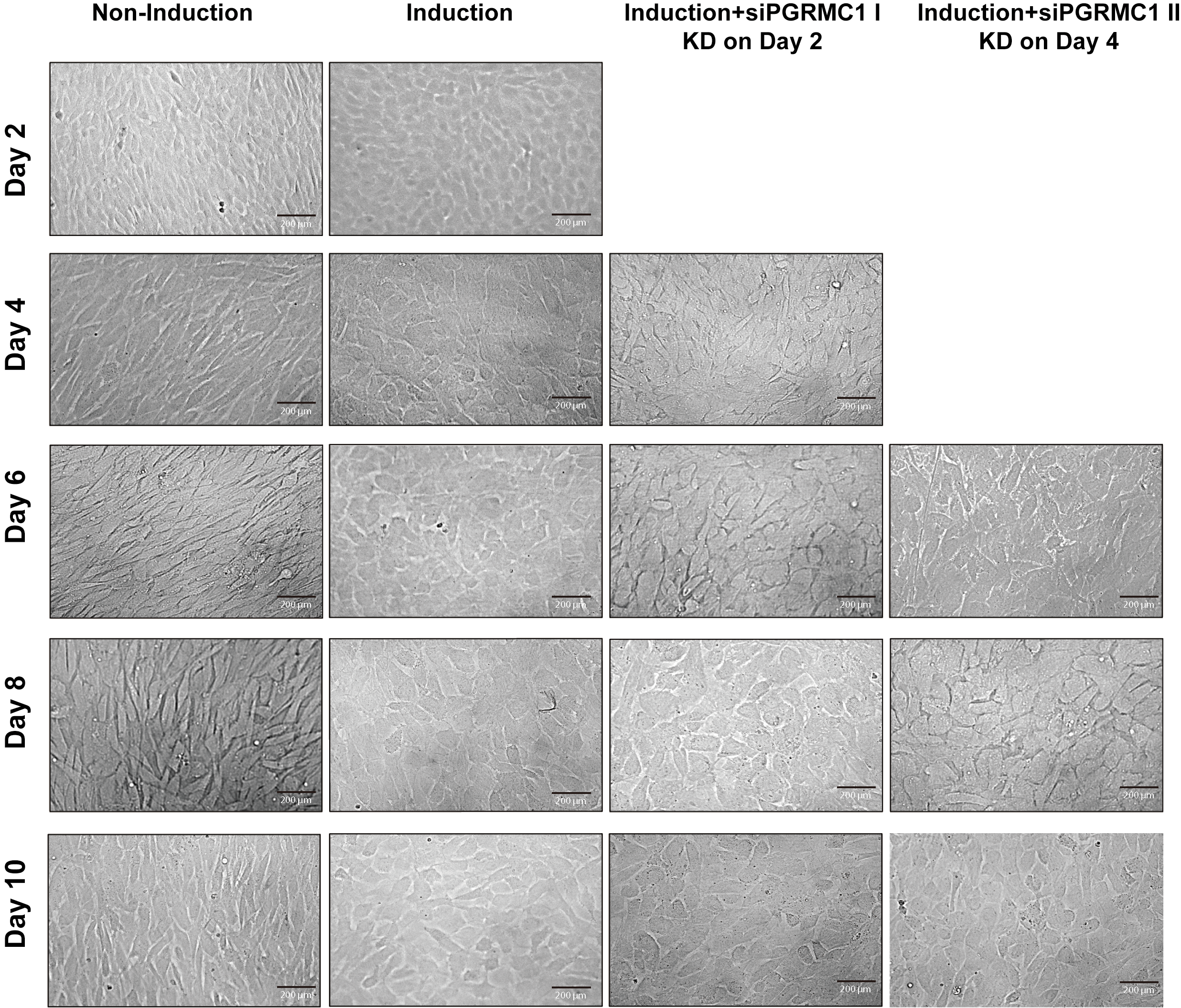

Supplement: Supplementary file 1 — Additional file 1: Supplementary Fig. 1. Rise-to-decline expression pattern of PGRMC1 is linked to the decidualization program. (A) PGRMC1 protein expression changes during 9 days of decidualization were measured by western blot in the St-T1 cell line. (B) PGRMC1 protein expression changes during 10 days of stimulation with MPA, cAMP, and DMSO, respectively, were measured by western blot in T-HESCs. (C) PGRMC1 protein expression levels on day 6 and day 10 when cultured with DMSO, nomegestrel (NOM), P4, cAMP, MPA/cAMP (M + A), and MPA, respectively, measured by western blot in T-HESCs. Supplementary Fig. 2. PGRMC1 is effectively downregulated by siRNA on protein level. (A) The PGRMC1 protein expression on day 2 and day 10 after transfection of T-HESCs with either 10 nM anti-PGRMC1 siRNA (siPGRMC1) or unspecific scrambled-control siRNA (siCTL). (B) A comparison of the PGRMC1 protein expression changes within 10 days after transfection of T-HESCs with either 10 nM siPGRMC1 or 10 nM siCTL. Supplementary Fig. 3. PGRMC1-downregulation before decidualization induction impairs morphological remodeling of T-HESCs. The cellular morphology changes of the T-HESCs induced with either DMSO (upper panel) or MPA/cAMP (down panel) after 10 days of siRNA treatment (siCTL, left panel; siPGRMC1, right panel). Scale bar: 200 µm. Supplementary Fig. 4. PGRMC1-downregulation after decidualization induction does not impair morphological remodeling of T-HESCs. The cellular morphology changes of the T-HESCs induced with either DMSO (non-induction, column 1) or MPA/cAMP (Induction, columns 2–4). I (column 3) and II (column 4) indicate that siRNA treatment on T-HESCs was conducted on day 2 or day 4 of decidualization induction, respectively. Scale bar: 200 µm. Supplementary Fig. 5. PGRMC1-downregulation after progestin induction does not impair decidualization in the St-T1 cell line. The mRNA expression levels of PGRMC1 (A, C) and PRL (B, D) in St-T1 treated with MPA/cAMP for decidualization in [file 12958_2024_1188_MOESM1_ESM.zip › Supplementary figure 4.tif]

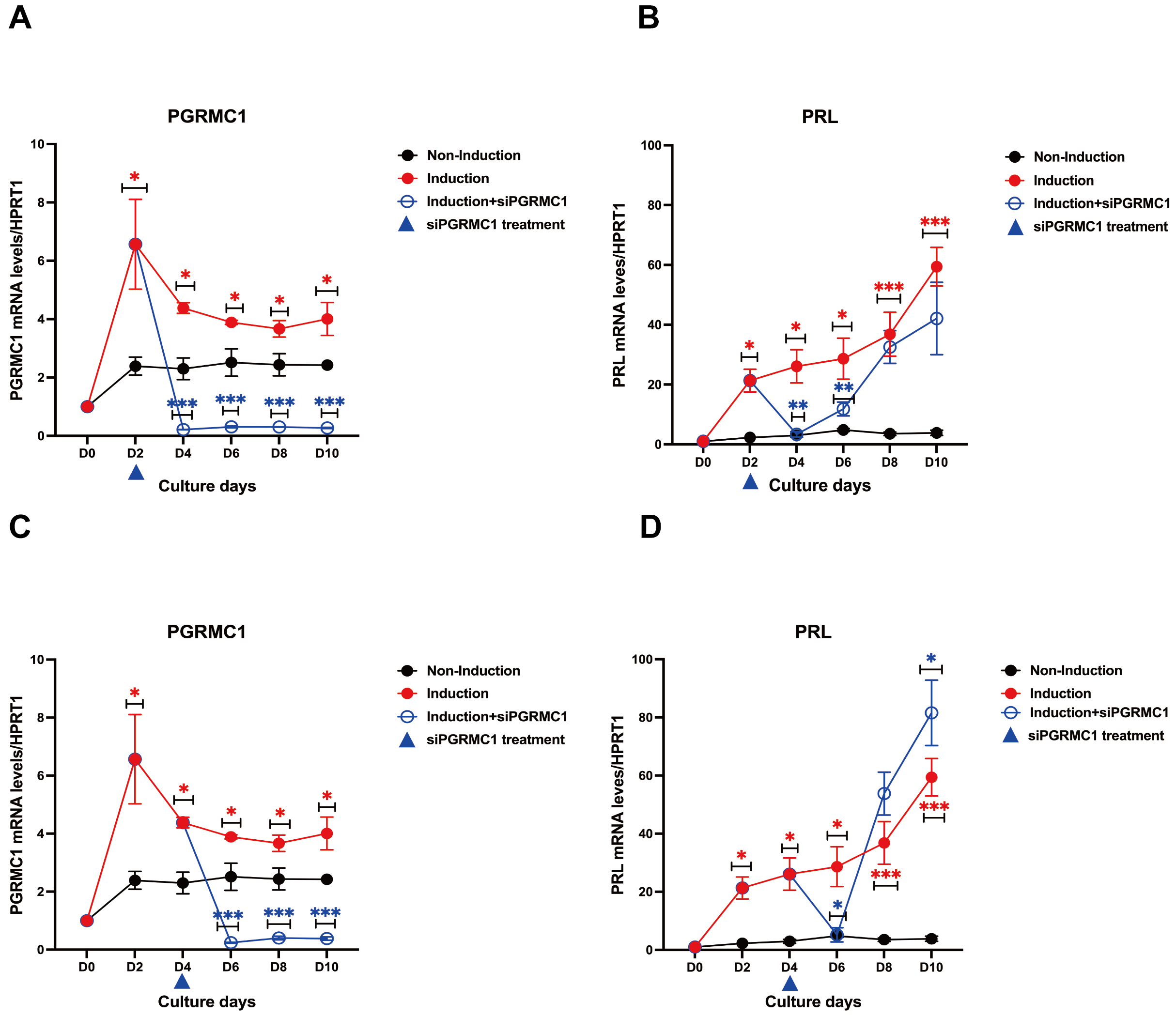

Supplement: Supplementary file 1 — Additional file 1: Supplementary Fig. 1. Rise-to-decline expression pattern of PGRMC1 is linked to the decidualization program. (A) PGRMC1 protein expression changes during 9 days of decidualization were measured by western blot in the St-T1 cell line. (B) PGRMC1 protein expression changes during 10 days of stimulation with MPA, cAMP, and DMSO, respectively, were measured by western blot in T-HESCs. (C) PGRMC1 protein expression levels on day 6 and day 10 when cultured with DMSO, nomegestrel (NOM), P4, cAMP, MPA/cAMP (M + A), and MPA, respectively, measured by western blot in T-HESCs. Supplementary Fig. 2. PGRMC1 is effectively downregulated by siRNA on protein level. (A) The PGRMC1 protein expression on day 2 and day 10 after transfection of T-HESCs with either 10 nM anti-PGRMC1 siRNA (siPGRMC1) or unspecific scrambled-control siRNA (siCTL). (B) A comparison of the PGRMC1 protein expression changes within 10 days after transfection of T-HESCs with either 10 nM siPGRMC1 or 10 nM siCTL. Supplementary Fig. 3. PGRMC1-downregulation before decidualization induction impairs morphological remodeling of T-HESCs. The cellular morphology changes of the T-HESCs induced with either DMSO (upper panel) or MPA/cAMP (down panel) after 10 days of siRNA treatment (siCTL, left panel; siPGRMC1, right panel). Scale bar: 200 µm. Supplementary Fig. 4. PGRMC1-downregulation after decidualization induction does not impair morphological remodeling of T-HESCs. The cellular morphology changes of the T-HESCs induced with either DMSO (non-induction, column 1) or MPA/cAMP (Induction, columns 2–4). I (column 3) and II (column 4) indicate that siRNA treatment on T-HESCs was conducted on day 2 or day 4 of decidualization induction, respectively. Scale bar: 200 µm. Supplementary Fig. 5. PGRMC1-downregulation after progestin induction does not impair decidualization in the St-T1 cell line. The mRNA expression levels of PGRMC1 (A, C) and PRL (B, D) in St-T1 treated with MPA/cAMP for decidualization in [file 12958_2024_1188_MOESM1_ESM.zip › Supplementary figure 5.tif]

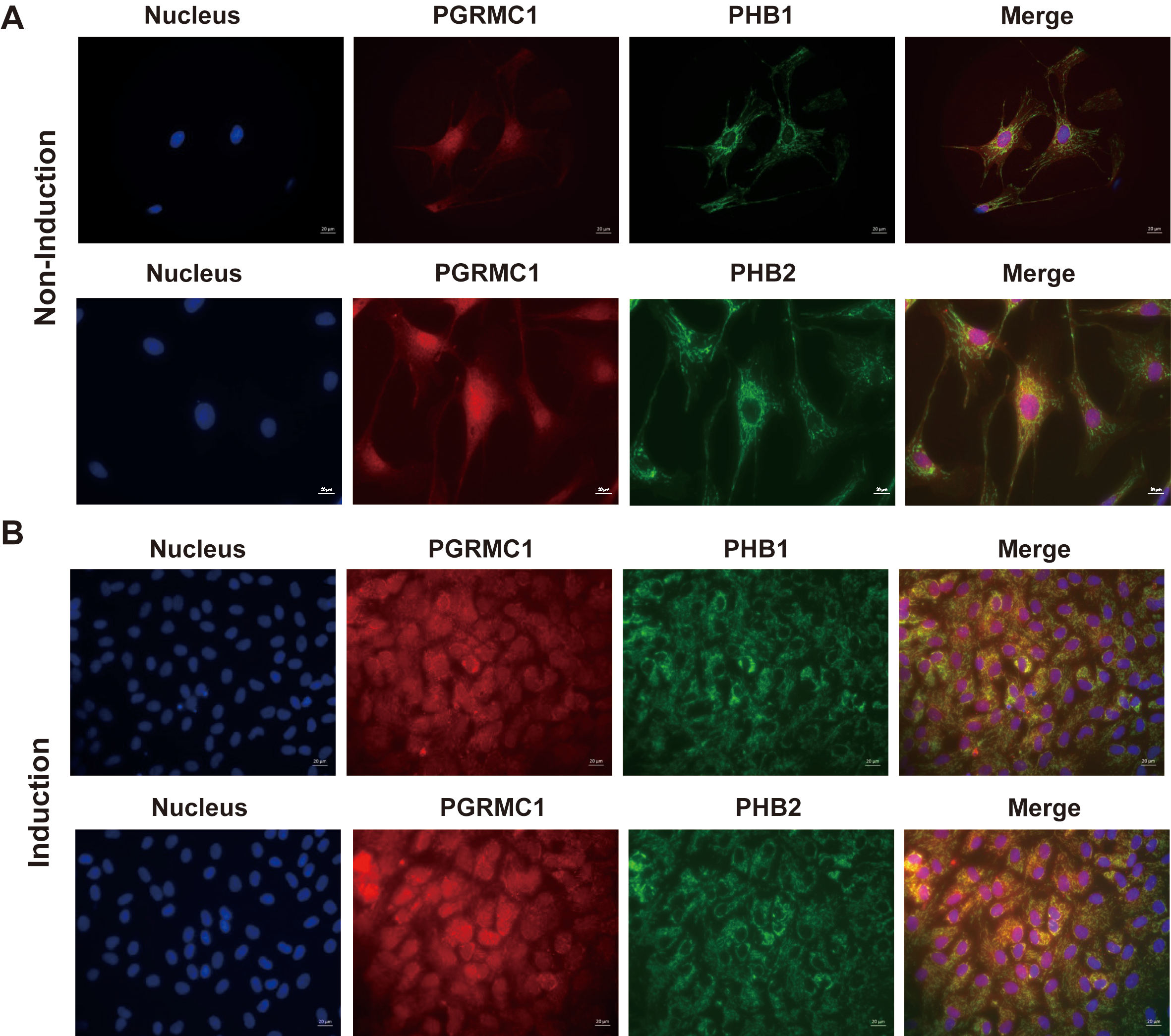

Supplement: Supplementary file 1 — Additional file 1: Supplementary Fig. 1. Rise-to-decline expression pattern of PGRMC1 is linked to the decidualization program. (A) PGRMC1 protein expression changes during 9 days of decidualization were measured by western blot in the St-T1 cell line. (B) PGRMC1 protein expression changes during 10 days of stimulation with MPA, cAMP, and DMSO, respectively, were measured by western blot in T-HESCs. (C) PGRMC1 protein expression levels on day 6 and day 10 when cultured with DMSO, nomegestrel (NOM), P4, cAMP, MPA/cAMP (M + A), and MPA, respectively, measured by western blot in T-HESCs. Supplementary Fig. 2. PGRMC1 is effectively downregulated by siRNA on protein level. (A) The PGRMC1 protein expression on day 2 and day 10 after transfection of T-HESCs with either 10 nM anti-PGRMC1 siRNA (siPGRMC1) or unspecific scrambled-control siRNA (siCTL). (B) A comparison of the PGRMC1 protein expression changes within 10 days after transfection of T-HESCs with either 10 nM siPGRMC1 or 10 nM siCTL. Supplementary Fig. 3. PGRMC1-downregulation before decidualization induction impairs morphological remodeling of T-HESCs. The cellular morphology changes of the T-HESCs induced with either DMSO (upper panel) or MPA/cAMP (down panel) after 10 days of siRNA treatment (siCTL, left panel; siPGRMC1, right panel). Scale bar: 200 µm. Supplementary Fig. 4. PGRMC1-downregulation after decidualization induction does not impair morphological remodeling of T-HESCs. The cellular morphology changes of the T-HESCs induced with either DMSO (non-induction, column 1) or MPA/cAMP (Induction, columns 2–4). I (column 3) and II (column 4) indicate that siRNA treatment on T-HESCs was conducted on day 2 or day 4 of decidualization induction, respectively. Scale bar: 200 µm. Supplementary Fig. 5. PGRMC1-downregulation after progestin induction does not impair decidualization in the St-T1 cell line. The mRNA expression levels of PGRMC1 (A, C) and PRL (B, D) in St-T1 treated with MPA/cAMP for decidualization in [file 12958_2024_1188_MOESM1_ESM.zip › Supplementary figure 6.tif]

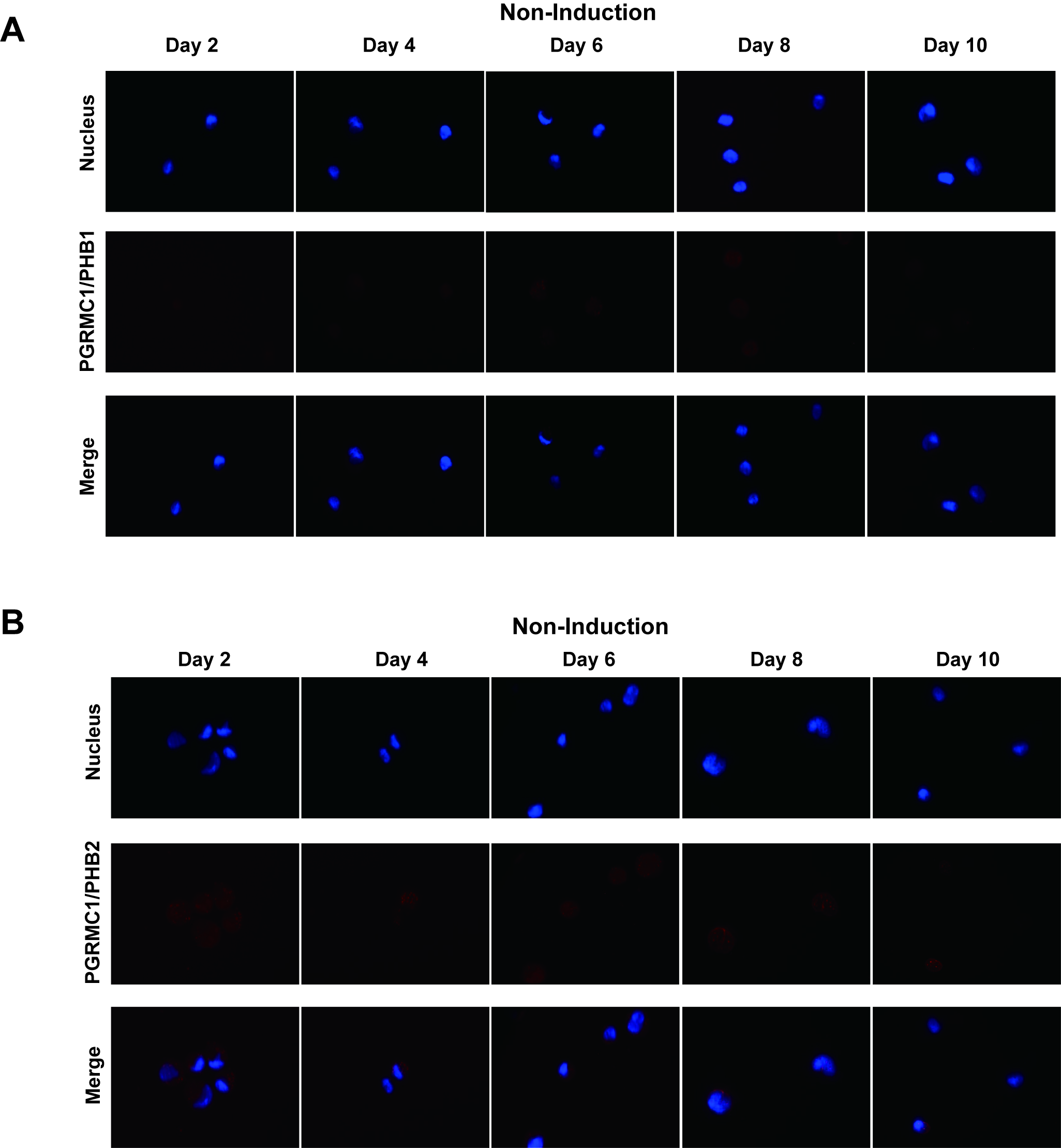

Supplement: Supplementary file 1 — Additional file 1: Supplementary Fig. 1. Rise-to-decline expression pattern of PGRMC1 is linked to the decidualization program. (A) PGRMC1 protein expression changes during 9 days of decidualization were measured by western blot in the St-T1 cell line. (B) PGRMC1 protein expression changes during 10 days of stimulation with MPA, cAMP, and DMSO, respectively, were measured by western blot in T-HESCs. (C) PGRMC1 protein expression levels on day 6 and day 10 when cultured with DMSO, nomegestrel (NOM), P4, cAMP, MPA/cAMP (M + A), and MPA, respectively, measured by western blot in T-HESCs. Supplementary Fig. 2. PGRMC1 is effectively downregulated by siRNA on protein level. (A) The PGRMC1 protein expression on day 2 and day 10 after transfection of T-HESCs with either 10 nM anti-PGRMC1 siRNA (siPGRMC1) or unspecific scrambled-control siRNA (siCTL). (B) A comparison of the PGRMC1 protein expression changes within 10 days after transfection of T-HESCs with either 10 nM siPGRMC1 or 10 nM siCTL. Supplementary Fig. 3. PGRMC1-downregulation before decidualization induction impairs morphological remodeling of T-HESCs. The cellular morphology changes of the T-HESCs induced with either DMSO (upper panel) or MPA/cAMP (down panel) after 10 days of siRNA treatment (siCTL, left panel; siPGRMC1, right panel). Scale bar: 200 µm. Supplementary Fig. 4. PGRMC1-downregulation after decidualization induction does not impair morphological remodeling of T-HESCs. The cellular morphology changes of the T-HESCs induced with either DMSO (non-induction, column 1) or MPA/cAMP (Induction, columns 2–4). I (column 3) and II (column 4) indicate that siRNA treatment on T-HESCs was conducted on day 2 or day 4 of decidualization induction, respectively. Scale bar: 200 µm. Supplementary Fig. 5. PGRMC1-downregulation after progestin induction does not impair decidualization in the St-T1 cell line. The mRNA expression levels of PGRMC1 (A, C) and PRL (B, D) in St-T1 treated with MPA/cAMP for decidualization in [file 12958_2024_1188_MOESM1_ESM.zip › Supplementary figure 7.tif]

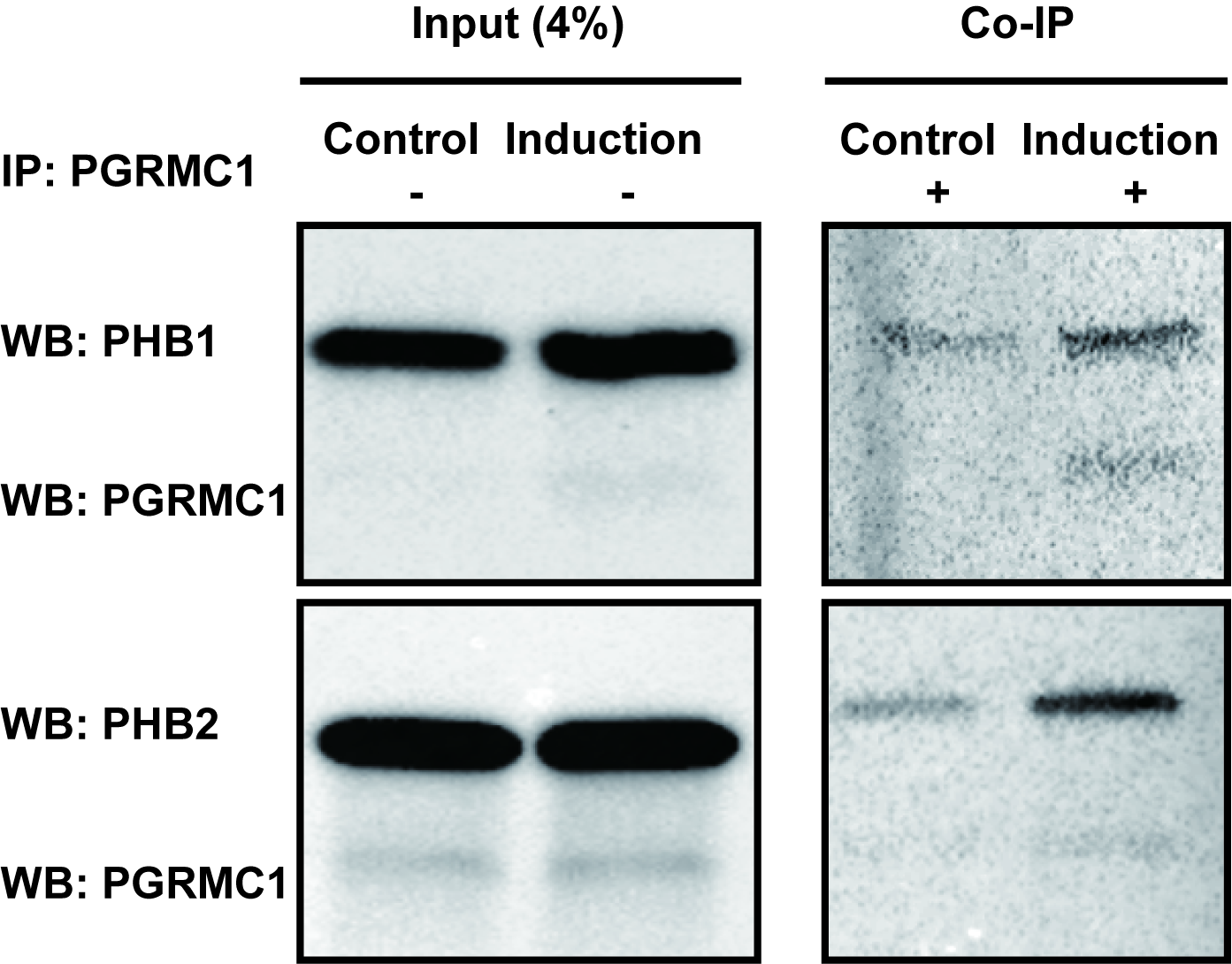

Supplement: Supplementary file 1 — Additional file 1: Supplementary Fig. 1. Rise-to-decline expression pattern of PGRMC1 is linked to the decidualization program. (A) PGRMC1 protein expression changes during 9 days of decidualization were measured by western blot in the St-T1 cell line. (B) PGRMC1 protein expression changes during 10 days of stimulation with MPA, cAMP, and DMSO, respectively, were measured by western blot in T-HESCs. (C) PGRMC1 protein expression levels on day 6 and day 10 when cultured with DMSO, nomegestrel (NOM), P4, cAMP, MPA/cAMP (M + A), and MPA, respectively, measured by western blot in T-HESCs. Supplementary Fig. 2. PGRMC1 is effectively downregulated by siRNA on protein level. (A) The PGRMC1 protein expression on day 2 and day 10 after transfection of T-HESCs with either 10 nM anti-PGRMC1 siRNA (siPGRMC1) or unspecific scrambled-control siRNA (siCTL). (B) A comparison of the PGRMC1 protein expression changes within 10 days after transfection of T-HESCs with either 10 nM siPGRMC1 or 10 nM siCTL. Supplementary Fig. 3. PGRMC1-downregulation before decidualization induction impairs morphological remodeling of T-HESCs. The cellular morphology changes of the T-HESCs induced with either DMSO (upper panel) or MPA/cAMP (down panel) after 10 days of siRNA treatment (siCTL, left panel; siPGRMC1, right panel). Scale bar: 200 µm. Supplementary Fig. 4. PGRMC1-downregulation after decidualization induction does not impair morphological remodeling of T-HESCs. The cellular morphology changes of the T-HESCs induced with either DMSO (non-induction, column 1) or MPA/cAMP (Induction, columns 2–4). I (column 3) and II (column 4) indicate that siRNA treatment on T-HESCs was conducted on day 2 or day 4 of decidualization induction, respectively. Scale bar: 200 µm. Supplementary Fig. 5. PGRMC1-downregulation after progestin induction does not impair decidualization in the St-T1 cell line. The mRNA expression levels of PGRMC1 (A, C) and PRL (B, D) in St-T1 treated with MPA/cAMP for decidualization in [file 12958_2024_1188_MOESM1_ESM.zip › Supplementary figure 8.tif]

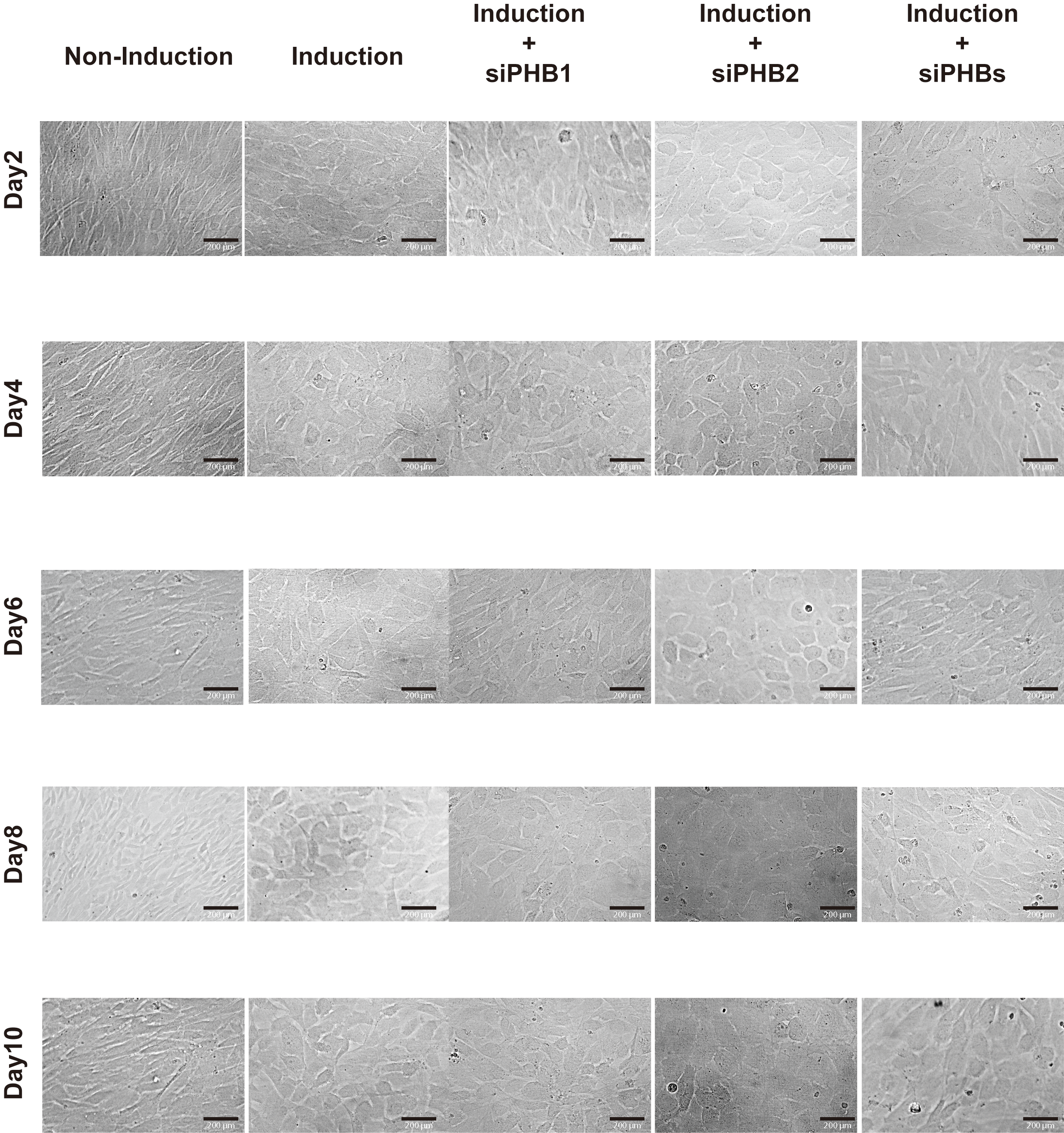

Supplement: Supplementary file 1 — Additional file 1: Supplementary Fig. 1. Rise-to-decline expression pattern of PGRMC1 is linked to the decidualization program. (A) PGRMC1 protein expression changes during 9 days of decidualization were measured by western blot in the St-T1 cell line. (B) PGRMC1 protein expression changes during 10 days of stimulation with MPA, cAMP, and DMSO, respectively, were measured by western blot in T-HESCs. (C) PGRMC1 protein expression levels on day 6 and day 10 when cultured with DMSO, nomegestrel (NOM), P4, cAMP, MPA/cAMP (M + A), and MPA, respectively, measured by western blot in T-HESCs. Supplementary Fig. 2. PGRMC1 is effectively downregulated by siRNA on protein level. (A) The PGRMC1 protein expression on day 2 and day 10 after transfection of T-HESCs with either 10 nM anti-PGRMC1 siRNA (siPGRMC1) or unspecific scrambled-control siRNA (siCTL). (B) A comparison of the PGRMC1 protein expression changes within 10 days after transfection of T-HESCs with either 10 nM siPGRMC1 or 10 nM siCTL. Supplementary Fig. 3. PGRMC1-downregulation before decidualization induction impairs morphological remodeling of T-HESCs. The cellular morphology changes of the T-HESCs induced with either DMSO (upper panel) or MPA/cAMP (down panel) after 10 days of siRNA treatment (siCTL, left panel; siPGRMC1, right panel). Scale bar: 200 µm. Supplementary Fig. 4. PGRMC1-downregulation after decidualization induction does not impair morphological remodeling of T-HESCs. The cellular morphology changes of the T-HESCs induced with either DMSO (non-induction, column 1) or MPA/cAMP (Induction, columns 2–4). I (column 3) and II (column 4) indicate that siRNA treatment on T-HESCs was conducted on day 2 or day 4 of decidualization induction, respectively. Scale bar: 200 µm. Supplementary Fig. 5. PGRMC1-downregulation after progestin induction does not impair decidualization in the St-T1 cell line. The mRNA expression levels of PGRMC1 (A, C) and PRL (B, D) in St-T1 treated with MPA/cAMP for decidualization in [file 12958_2024_1188_MOESM1_ESM.zip › Supplementary figure 9.tif]
